# Boosting wisdom of the crowd for medical image annotation using training performance and task features

**DOI:** 10.1186/s41235-024-00558-6

**Published:** 2024-05-20

**Authors:** Eeshan Hasan, Erik Duhaime, Jennifer S. Trueblood

**Affiliations:** 1 Department of Psychological and Brain Sciences, Indiana University, 1101 E. 10th St., Bloomington, IN 47405-7007 USA; 2 Cognitive Science Program, Indiana University, Bloomington, USA; 3 Centaur Labs, Boston, USA

## Abstract

A crucial bottleneck in medical artificial intelligence (AI) is high-quality labeled medical datasets. In this paper, we test a large variety of wisdom of the crowd algorithms to label medical images that were initially classified by individuals recruited through an app-based platform. Individuals classified skin lesions from the International Skin Lesion Challenge 2018 into 7 different categories. There was a large dispersion in the geographical location, experience, training, and performance of the recruited individuals. We tested several wisdom of the crowd algorithms of varying complexity from a simple unweighted average to more complex Bayesian models that account for individual patterns of errors. Using a switchboard analysis, we observe that the best-performing algorithms rely on selecting top performers, weighting decisions by training accuracy, and take into account the task environment. These algorithms far exceed expert performance. We conclude by discussing the implications of these approaches for the development of medical AI.

**Supplementary Information:**

The online version contains supplementary material available at 10.1186/s41235-024-00558-6.

## Introduction

The future of medical artificial intelligence (AI) relies on the existence of large, high-quality labeled biomedical image datasets for machine learning training (Ørting et al., 2020; Codella et al., 2019; Tschandl et al., 2018). Currently, the lack of such datasets is considered one of the largest bottlenecks in the development and training of medical AI systems (Ørting et al., 2020; Kentley et al., 2023; Duhaime et al., 2023). Traditionally, these datasets have been meticulously curated based on the consensus of expert medical professionals (Tschandl et al., 2018; van der Wal et al., 2021). In contrast, the labeling of datasets involving everyday objects, such as ImageNet, scales easily through the use of online crowdsourcing (Deng et al., 2009). Thus, some researchers and entrepreneurs have suggested that labeling medical images through crowdsourcing might provide one solution to the medical AI data bottleneck (Ørting et al., 2020; Alialy et al., 2018; Kentley et al., 2023; Duhaime et al., 2023).

Applying crowdsourcing to complex medical image decision-making tasks presents distinct challenges (Tucker et al., 2019). Not only are the images and tasks often unfamiliar to individuals outside the medical specialization, but they often need to be classified into one of many different classes with subtle differences. Even experts with extensive training are often wrong (Tschandl et al., 2019; Kämmer et al., 2017; Barnett et al., 2019). In this high-stakes domain, training medical AI systems with low-quality datasets could have serious health impacts.

Effectively harnessing collective intelligence using the wisdom of the crowd approaches has emerged as a powerful approach to solving many complicated classification problems including misinformation (Allen et al., 2021) and deep fake detection (Groh et al., 2022) as well as medical image decision-making (Kurvers et al., 2016; Hasan et al., 2023; Duhaime et al., 2023). In medical domains, the aggregated decisions of multiple individuals can, not only match but, at times, surpass the performance of seasoned medical experts (Hasan et al., 2023; Kurvers et al., 2016; Litvinova et al., 2022; Duhaime et al., 2023). In this paper, we explore the possibility of harnessing collective wisdom from a wide variety of individuals to obtain classification decisions on complex medical images of skin lesions.

Translating the wisdom of the crowds from a controlled lab environment to a real-world application requires the testing and development of scalable systems that can acquire a large number of decisions in a short time at low costs (Kentley et al., 2023; Ørting et al., 2020; Duhaime et al., 2023). A company—Centaur Labs—developed an app-based platform where individuals with a varying range of experience and expertise sign up to provide medical decisions (Press, 2021; Duhaime et al., 2023). This is provided as a service to medical institutions that are interested in harnessing the wisdom of the crowd to label large medical datasets. In this paper, we use data collected by Centaur Labs in Duhaime et al. (2023), to comprehensively test the effectiveness of different approaches at arriving at the group decision.

Participants made decisions on images from the International Skin Lesion Collaboration (2018) (Codella et al., 2019; Tschandl et al., 2018). The images were collected from several different institutions so that they contained a wide variety of skin types and lesions (Tschandl et al., 2018). Participants categorized skin lesion images into one of seven different classes. Participants received feedback on their decisions and learned the task after they signed up on the app. This task was difficult since even board-certified dermatologists made mistakes and had an accuracy of $74.7\%$ (Tschandl et al., 2019). Further, the true label of the lesion was often determined through extensive testing such as histopathology and microscopy (Tschandl et al., 2018). Hence, all the necessary information about the true label of the lesion was sometimes not knowable through the image alone.

Since individuals could freely sign up on the mobile app, they had a range of different backgrounds. Many of them were medical students or pre-med students whereas others had no medical experience. As a result, there was a large variation in the accuracy, prior information, and dermatology knowledge across individuals. When confronted with a wide range of individuals, what is the best way of combining their individual decisions to produce a high-quality labeled dataset? On the one hand, the wisdom of the crowd crucially hinges on collecting enough decisions so that the biases of an individual decision-maker are canceled out during the aggregation process. The diversity of the crowd is an important component in the success of the wisdom of the crowd (Davis-Stober et al., 2014; Surowiecki, 2005; Broomell and Davis-Stober, 2023). Using the most common decision as the group decision—the majority-plurality rule—has been shown to be very robust and easy to implement (Hastie and Kameda, 2005; Duhaime et al., 2023). Weighting individual decisions (e.g., by their accuracy on training images) might have limited effectiveness since unweighted aggregation can perform as well as more complicated algorithms (Collins et al., 2023; Armstrong, 2001; Clemen, 1989). On the other hand, when there is a large dispersion in individual performance, it is possible to exploit the dispersion to improve the crowd performance (Mannes et al., 2014; Budescu and Chen, 2015; Duhaime et al., 2023).

On the app, the training dataset was used to assess the performance of participants and give them daily rewards. This gave us the opportunity to objectively measure the performance of individuals. How does one effectively use this information to design wisdom of the crowd algorithms? In one approach, one could select the best decision-makers and discard the individuals with low accuracy. In another approach, one could weigh the decisions by performance. Or one could select the top performers and weigh their decisions appropriately.

The first approach based on selecting a smaller smarter sub-crowd has shown to improve accuracy in some domains (Atanasov and Himmelstein, 2023; Mannes et al., 2014; Galesic et al., 2018; Goldstein et al., 2014; Budescu and Chen, 2015; Afflerbach et al., 2021). While different measures can be used to select a smarter sub-crowd (Atanasov and Himmelstein, 2023), we can select individuals based on task performance on the training images. However, it is not clear how many people one must retain during the aggregation process. Tetlock and Gardner (2016) and Himmelstein et al. (2023) argue for the existence of superforecasters, who if identified can consistently beat the crowd. In Goldstein et al. (2014), the authors find a decreasing relationship with the number of experts, where the performance decreases as more individuals are included. However, including the decisions of more than one expert turned out to be useful. On the other hand, relying on one expert makes the algorithm susceptible to biases and noise of the expert. Despite their extensive training, even experts are susceptible to noise in their decision process (Kahneman et al., 2021; Hasan and Trueblood, 2022; Hasan et al., 2023; Koriat, 2012; Goldstein et al., 2008; Litvinova et al., 2022; Kurvers et al., 2023) and might even make inconsistent decisions on the same image (Hasan et al., 2023, 2022; Litvinova et al., 2022). Hence, there seems to be a need to not just rely on one expert but take multiple readings to reduce the noise in the final decision. This indicates that decisions might be improved by aggregating the decisions of multiple people.

The second approach is to apply weight to every individual’s decision based on their performance (Collins et al., 2023; Atanasov et al., 2017; Armstrong, 2001; Budescu and Chen, 2015; Wang et al., 2011, 2011b; Duhaime et al., 2023). Initial results in Duhaime et al. (2023) showed that directly weighting by the training accuracy can improve test accuracy. However, there are different ways in which performance is measured and weighted (Collins et al., 2023). For example, suppose we are interested in using accuracy as a means of measuring performance. It is unclear how this accuracy score is converted into a weight. For a binary classification problem with two classes of equal prevalence, a person responding randomly will have an accuracy of 0.5. Should one then assign the weight to be 0 for that individual? Should one transform it by some function—say the log before aggregation?

One might perform weighting in such a way as to account for individual biases and idiosyncrasies of individual decision-makers (Juni and Eckstein, 2017; Steyvers et al., 2014). For instance, an individual might have a tendency to be cautious when declaring a skin lesion as cancerous. On the other hand, another individual might err on the other side and call a lesion cancerous even when there is a small but non-zero chance of it being cancerous (Wickens, 2001). Some wisdom of the crowd algorithms based on signal detection theory re-calibrate the judgments of different individuals before aggregating to account for these biases (Steyvers et al., 2014; Juni and Eckstein, 2017). In this paper, we will address the question of whether one should correct for differences in response tendencies and accuracy in multiclass classification tasks.

We adopt a comprehensive approach and develop models of different sophistication. First, we establish a baseline using simple voting (i.e., majority voting), where the decision of the crowd is determined by the majority decision on every image. We compare this to algorithms based on selection alone while varying the number of individuals that are selected. We test simple models based on directly weighting by training accuracy. We develop a Bayesian framework that is based on estimating the probability of the different classes. Using this framework, we specify different models that can account for individual differences in accuracy. We also develop algorithms that are based on the pattern of errors that are made in the task. We then tailor these for individuals by explicitly taking into account the different response tendencies. We also take into account the different prevalence rates of different lesion types. Finally, we conduct a comprehensive switchboard analysis, varying all of the different factors that make the algorithms (Zhao et al., 2022; Turner et al., 2018).

## Methods

We used an app-based platform to recruit participants. The task involved the classification of images of skin lesions from the International Skin Imaging Collaboration (ISIC) 2018 Challenge (Codella et al., 2019; Tschandl et al., 2018). The goal was to obtain decisions on the 1511 test images to investigate the effectiveness of the wisdom of the crowds and to study different aggregation algorithms in a medical setting. We use the same data as Duhaime et al. (2023) for our analysis.

### Participants

Participants were recruited from an iOS app-based platform called DiagnosUs. Participants were told that they could improve their skills and contribute to medical artificial intelligence. They were rewarded based on their daily performance. The daily prizes were $40, $25, $20, $15, $10, $5, $4, $3, $2, and $1, respectively. The winners were determined based on their performance on the training set. To win a prize, they would have needed to contribute at least 100 decisions on that day. The data were collected for 14 days. Participants agreed to the terms of service agreement where they consented to their data being used for commercial and academic purposes. Subsequent data analysis of the collected data was approved by the Institutional Review Board at Indiana University Bloomington (#20135).

Of the 458 people that signed up on the app, 315 participants gave at least one response in the task. In terms of gender, 167 (53.0%) of the participants identified as female, 127 (41.0%) as male, 8 (2.5%) as other, and 13 (4.1%) gave no response. There was a large variation in the geographical location, experience, and occupation of the participants. Individuals from all over the world belonging to 47 countries participated. Most of them (124, 39.4%) were from the Americas. Eighty-seven (27.6%) were from Africa, 50 (15.9%) were from Asia, and 39 (12.4%) were from Europe. Most (64.7%) of the participants said that they had no dermatology experience while others had differing amounts of dermatology experience (16.1%<1 year; 7.4% 1-3 years; 2.4% 3-5 years; 1.2% 5-10 years; 1.8% 10+ years, 6.1% no response). A large number of participants were medical students (56.5%) or pre-medical students (8.8%). Some individuals were residents or fellows (4.3%), attending physicians (4.0%), nurse practitioners (2.1%), and 10.3% said that they had no medical experience. Some respondents (6.1%) gave no response to this question.

### Materials

The images were from the International Skin Imaging Collaboration (ISIC) 2018 Challenge (Codella et al., 2019; Tschandl et al., 2018). The full details of the dataset and challenge can be found on the website (https://challenge.isic-archive.com/landing/2018/47/) and in Tschandl et al. (2018). We go over the main details here. These skin lesion images were obtained from a historical sample of patients from several different institutions for skin cancer screening. The true label of the dataset for malignancy was obtained using histopathology. The true label of the dataset for non-malignancy was determined through one of the following methods—histopathology, reflectance confocal microscopy, expert consensus, and observation in follow-up visits (Tschandl et al., 2018). That is, the lesion did not change during digital dermatoscopic follow-up over two years with at least three images. The images were collected so that they reflected a large variation in the kind of skin types, imaging techniques, and lesions.

The dataset was divided into 7 different types of skin lesions—actinic keratosis (AKIEC), benign keratosis (BKL), basal cell carcinoma (BCC), dermatofibroma (DF), melanocytic nevi (NV), melanoma (MEL), and vascular lesions (VASC). MEL and BCC are cancerous, AKIEC is precancerous, while NV, DF, and VASC are non-cancerous. The data collected were subdivided into 10015 train images and 1195 test images. In an effort to diversify the images, an additional 316 images was added to the test set. Hence, there was a total of 1511 test images by Tschandl et al. (2018). The labels for the test set were obtained by contacting the authors of Tschandl et al. (2018) after data collection. The distribution of the images was skewed as shown in Table 1, with most of them belonging to the two dominant classes—NV and MEL. As shown in Table 1, there were more benign cases in the dataset compared to malignant, which was reflective of the real world (Tschandl et al., 2018). However, compared to the real world, the number of malignant cases was over-represented in the dataset (Tschandl et al., 2018).

**Table 1:** Distribution of images based on their type from ISIC (2018)

Lesion type	Abbreviation	No. of train images	Percentage of total (Train) (%)	No. of test images	Percentage of total (test) (%)
Actinic Keratosis	AKIEC	327	3.5	43	2.8
Basal Cell Carcinoma	BCC	514	5.5	93	6.2
Benign Keratosis	BKL	1099	11.8	217	14.4
Dermatofibroma	DF	115	1.2	44	2.9
Melanoma	MEL	1113	11.9	171	11.3
Melanocytic Nevi	NV	6705	64.5	908	60.1
Vascular Lesion	VASC	142	1.5	35	2.3
Total	–	10015	100	1511	100

### Procedure

Participants first signed up for the app and provided their demographic information. After this, they could do an optional short tutorial block. In the main task, participants saw a single image on each trial and had to classify it into one of the seven different classes. Participants saw a 20-second timer within which they had to classify the image. Responses with response times longer than 20 s or with invalid response times were discarded as a part of the data-cleaning pipeline (0.8% responses). The average time to classify an image was 8.5 sec. Images were randomly sampled from the train and test sets.

The images from the train set were sampled such that they were counterbalanced across the seven classes. The images from the test set were randomly sampled and hence were not counterbalanced across the seven classes. Participants were not told whether the image belonged to the train or the test set at the beginning of the trial. The image belonged to the train set $75\%$ of the time and test set $25\%$ of the time. If the image belonged to the train set, they received feedback on the trial. If the image belonged to the test set, they did not receive feedback on the trial. Participants could label images for as long as they liked. They needed to label at least 100 images to be entered into the daily competition. They could exit the app at any time and could resume when they wanted to.

## Behavioral results

We now present the behavioral results. The results of the different aggregation algorithms will be described and presented in the next section.

### Overview of dataset

**Fig. 1:**
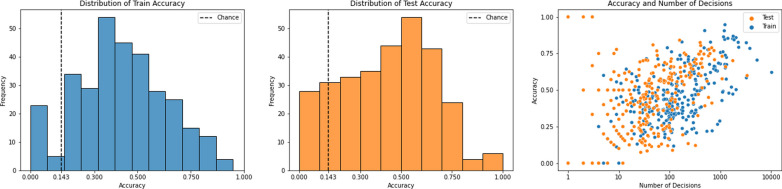
The panels on the left and the middle show the distribution of mean accuracy of different individuals for the test and train images, respectively, across all images. The chance accuracy is calculated as 1/7 since there were 7 different classes. The panel on the right shows the relationship between the accuracy of an individual and the number of responses provided by the individual

A total of 143209 decisions were made in the task. Of these, 107506 decisions were made on training images and 35703 decisions on the test set. Each participant participated for a median of 2 days (Mean: 3.5; IQR: 1–4; Max: 14) and contributed a median of 100 (Mean: 130.4; IQR: 31–121; Max: 4, 218) decisions per day. Across the 14 days, they saw a median of 135 images (Mean: 454.6, IQR: 33.5–395; Max 13, 563). As shown in Fig. 1 and Table 2, there was a large skew in the number of responses with a few individuals contributing a disproportionately large number of responses. For instance, 60 individuals made more than 500 decisions across the 14 days. These 60 individuals make up about $19\%$ of all individuals who participated in the experiment and contributed $76.6\%$ of responses. A single individual made more than 10000 decisions, which made up $9.5\%$ of the data set.

When analyzed at the image level, there was a large difference in the number of total decisions on train and test images. On the training set, there was a median of 3 responses per image with a large range in the number of responses (IQR: 1–13; Min: 1; Max= 156). On the test set, there was a median of 24 responses per image. Since the experiment was designed so that each of the test images had a similar number of responses, we observed a narrower range (IQR: 23–24; Min= 21; Max= 25).

**Table 2:** This table shows the number of responses contributed by participants as well as the mean train and test accuracy for the data contributed by them

	No. of train decisions	No. of individuals	Perc. of individuals (%)	Perc. of decisions (%)	Mean train accuracy (%)	Mean test accuracy (%)
0	0–100	154	48.89	4.85	37.54	35.19
1	100–500	109	34.60	22.08	48.78	46.50
2	500–1000	20	6.35	13.54	56.79	55.41
3	1000–3000	26	8.25	35.47	71.31	65.43
4	3000–5000	3	0.95	9.57	73.33	65.59
5	5000–10,000	1	0.32	4.90	70.45	70.07
6	10,000–1,000,000	1	0.32	9.49	62.08	59.89

### Accuracy

As shown in Fig. 1, participants’ average accuracy for the training data set was $41.6\%$ (IQR: 28.2%–57.1%) and the testing set was $42.7\%$ (IQR: 24.4%–60.7%). This indicated that most participants performed the task with better accuracy than chance. However, this was much lower than the average accuracy of dermatologists of $74.7\%$ (70.8%–78.6%) (Tschandl et al., 2019). We also note the wide distribution of accuracy of the participants in our dataset.

As shown in Fig. 1, the log of the number of decisions that individuals contributed was positively correlated with their accuracy; $r(313)=.67$ $(p<0.001)$ for the train set and $r(300)=.39$ $(p<0.001)$ for the test set. Hence, the more accurate individuals provided a larger number of decisions. The average accuracy of the train set was $61.2\%,$ and test set was $58.1\%$. When the average accuracy of the data is calculated, and not at the individual level, the accuracy shifts closer to the accuracy of the individuals who contributed more responses. Since these individuals were also the more accurate ones, the average accuracy of the data is higher than the participants’ average accuracy reported above.

**Fig. 2:**
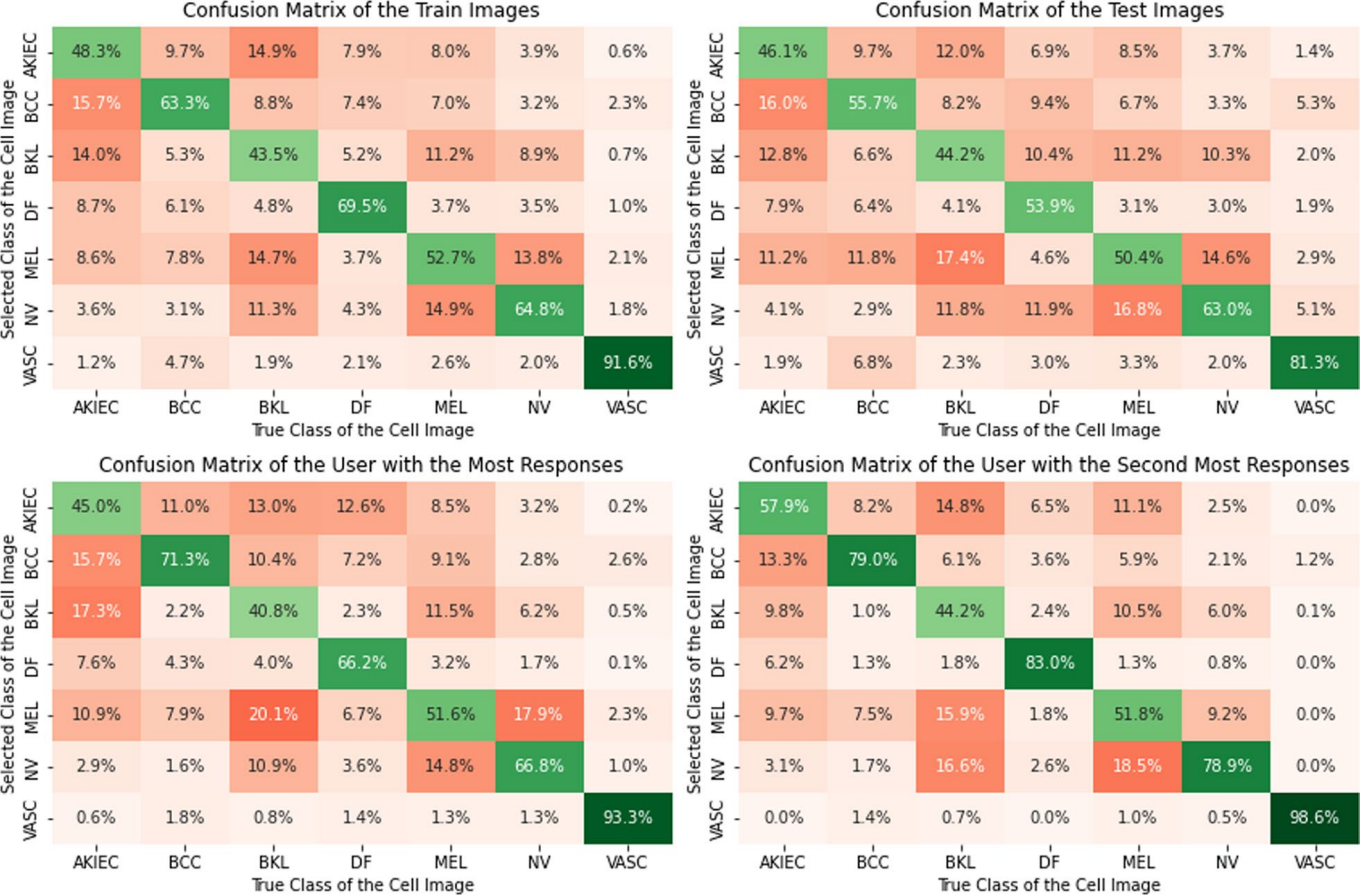
The top two panels show the confusion matrices when we pool the decisions from all individuals for the test and the train set, respectively. The bottom two panels show the confusion matrices for training for the individuals that provided the most and second most responses on the train set

We calculated the accuracy based on the lesion type. As observed in Fig. 2, there was a large difference in the performance across the lesion types. Consider the panel on the top left. This shows the confusion matrix for the training images. For example, participants were pretty good at identifying VASC and correctly identified it $91.6\%$ of the time. Comparatively, participants were not very good at identifying AKIEC and identified it $48.3\%$ of the time. We also observed that the types of errors were not random. For instance, NV was misclassified as MEL $13.8\%$ of times but only $3.9\%$ as AKIEC. We note that the confusion matrix for the train and test images was similar, but there were notable differences. For instance, the test set had elevated misses for low-frequency classes such as VASC and DF as compared to the train set. This pattern of errors is similar to low prevalence effects documented in other medical image domains (Wolfe et al., 2005; Trueblood et al., 2021).

In the lower two panels, we compared the confusion matrices on the training data for the two participants with the most number of responses. While we note that the patterns of mistakes were similar, there were also some differences. For example, the participant with the second most number of responses also had a higher accuracy across all of the lesion types and overall made fewer errors. We also note that for instance, they correctly identify a similar number of MEL as the first participant ($51.6\%$ compared to $51.8\%$) but they do so at the cost of misidentifying $17.9\%$ as opposed to $9.2\%$ of NV as MEL.

## Discussion

We make the following observations. First, a substantial imbalance exists in participant responses, wherein a small number of individuals contribute a disproportionate quantity of responses. Second, those who provide a greater number of responses tend to also exhibit higher levels of accuracy. Third, we observe individual differences in performance, characterized by a diverse range of accuracy scores. Fourth, confusion matrices of lesion types indicate varying frequencies of specific errors. Fifth, although shared errors are observable across different individuals, individual differences in patterns of errors are also apparent.

We note that designing algorithms that address the substantial individual differences in accuracy and response patterns might be crucial to optimally aggregating decisions for the wisdom of the crowd approaches. Accounting for individual differences in accuracy where the decisions of more accurate individuals are given more weight might lead to higher accuracy in the crowd decision. Further, accounting for the specific patterns of errors of different individuals might help appropriately weight decisions for the different lesion types and lead to optimal use of information in each response.

## Modeling methods

In this paper, the goal was to comprehensively test different ways of arriving at the group decision when soliciting individual decisions from an app-based interface. We build on the two simple methods tested in Duhaime et al. (2023) and comprehensively test different aggregation approaches. Specifically, we examine two types of aggregation approaches. First, we try selection where the responses from a set of top-performing individuals are used while others are discarded. Second, we examine weight approaches, where we weigh individuals based on some function of their accuracy. Finally, we will also try hybrid approaches that combine selection and weighting.

We use the following notation throughout the paper. Let the set of lesion types be $\mathbb {T}$. Let the 7 different lesion types AKIEC, BCC, BKL, DF, MEL, NV and VASC be $T_1,T_2,.. T_7 \in \mathbb {T}$. Our goal is to define the weights for each of the decisions for the 7 different lesion types.

Different individuals saw different images and made decisions about them. Since we are aggregating decisions on a given test image, we define the crowd $C_i$ in terms of the *i*th test image. Suppose participants $P_1, P_2,... P_n$ have made decisions $d_{i,1}, d_{i,2}... d_{i,n}$ on the *i*th test image to form the crowd $C_{i}$. Hence, $C_{i}$ is a set of decisions on *i*.

We define a weight function $w_{T}(d_{i,j})$ which is a function of the individual decision $d_{i,j}$ - for each lesion type $T_{k}$. The decision of the crowd $D_i$ on the *i*th test image is obtained by summing these weights for the decisions $d_{i,j}$ that are a part of the crowd $C_i$ and selecting the type with the largest weight.

$$\begin{aligned} D_{i}= \underset{T \in \mathbb {T}}{\text {argmax}} \sum _{d_{i,j} \in C_i}w_{T}(d_{i,j}) \end{aligned}$$

In this paper, we first use the simple voting algorithm to establish a baseline. We then test two different methods of aggregating decisions. We first test algorithms where individuals are selected based on their training accuracy and then we test algorithms based on weighting decisions by training accuracy. Finally, we conduct a switchboard analysis where we test hybrid algorithms that combine both selection and accuracy weighting.

### Simple voting

The baseline algorithm that we consider is the majority-plurality rule (Hastie and Kameda, 2005; Duhaime et al., 2023) or simple voting. This is the simplest algorithm where we retain all the decision-makers that form the crowd $C_{i}$ and give an equal weight of 1 to each of their votes.

$$\begin{aligned} w_{T}(d_{i,j})= {\left\{ \begin{array}{ll} 1\ \text {if}\ d_{i,j}=T \\ 0\ \text {if}\ d_{i,j} \ne T \end{array}\right. } \end{aligned}$$

### Algorithms based on selection weighting

In this section, we describe the first set of algorithms that are based on selecting individuals based on their training performance. Individuals that are not selected are discarded during the aggregation process by setting their weight to zero. These algorithms are based on the idea that excluding participants with poor judgment improves the quality of the crowd and hence the accuracy (Mannes et al., 2014; Goldstein et al., 2014).

We define a subset $S_i$ of $C_i$ which is a subset of decisions made on the *i*th test image. We define the weights using the indicator function ${\textbf {1}}_{S_i}$. That is, if the decision $d_{i,j}$ is in the subset of decisions $S_i$, then the weight is 1, else it is 0. If the subset $S_i$ includes everyone that has made a decision on the image *i*, then $S_i = C_i$ and is equivalent to simple voting.

$$\begin{aligned} w_{T}(d_{i,j})= {\left\{ \begin{array}{ll} {\textbf {1}}_{S_i}\ \text {if} \ d_{i,j}=T\\ 0\ \text {if} \ d_{i,j} \ne T \end{array}\right. } \end{aligned}$$

In this paper, we use the training accuracy of the individuals that made decisions $d_{i,j}$ in $C_i$ to define $S_i$. Let the training accuracy of the $j$th individual that made decision $d_{i,j}$ in $C_i$ be $a_j$. Let $M_{r,i}$ be the set of top *r* decisions made by individuals with the highest training accuracy who decided in $C_i$. Since on every test image, we had between 21 and 25 decisions, we vary *r* from 1 to 20. That is, we set $S_i=M_{ri}$ and vary *r* from 1-20. Thus, there was an equal number of decisions on each image.

### Algorithms based on accuracy weighting

In this section, we describe the different algorithms that rely on weighting the decisions made by individuals based on their training performance. In this paper, we consider several different approaches to weighting decisions.

#### Simple accuracy weighting (SAW)

The weight of each individual decision is the training accuracy of the participant that made the decision, which is calculated as the fraction correct on the train data (Duhaime et al., 2023). This includes the decisions made on all of the 7 different types of skin lesions. Let the training accuracy of the *j*th participant $P_j$ be $a_j$. In this algorithm, we summarize the performance of each participant using a simple metric that we use to weigh the decision.

$$\begin{aligned} w_{T}(d_{i,j})= {\left\{ \begin{array}{ll} a_j\ \text {if}\ d_{i,j}=T\\ 0\ \text {if}\ d_{i,j} \ne T\\ \end{array}\right. } \end{aligned}$$

#### Bayesian-log accuracy weighting (LAW)

Our goal is to estimate the probability that an image is of type *T* given decisions $d_{i,j}$. Let $D_{i,j}$ be the random variable that encodes the decision of the *j*th person on the *i*th image. Using Bayes theorem for the first equality and independence assumption for the second equality, we have

$$P(T \mid D_{i,1}=d_{i,1}\text { and }D_{i,2} = d_{i,2}) = \frac{P(D_{i,1}= d_{i,1}\text { and }D_{i,2}=d_{i,2} \mid T) P(T)}{P(D_{i,1}= d_{i,1}\text { and }D_{i,2}=d_{i,2})}$$

$$\begin{aligned} = \frac{P(D_{i,1}=d_{i,1} \mid T)P(D_{i,2}=d_{i,2} \mid T) P(T)}{P(D_{i,1}=d_{i,1}\text { and }D_{i,2}=d_{i,2})} \end{aligned}$$.

The ratio of the probability for two types $T_1$ and $T_2$ is given as follows:

$$\begin{aligned} \frac{P(T_1 \mid D_{i,1}=d_{i,1}\text { and }D_{i,2} =d_{i,2})}{P(T_2 \mid D_{i,1}=d_{i,1}\text { and } D_{i,2}=d_{i,2})} = \frac{P(D_{i,1}=d_{i,1} \mid T_1) P(D_{i,2}=d_{i,2} \mid T_1) P(T_1)}{P(D_{i,1}=d_{i,1} \mid T_2) P(D_{i,2}=d_{i,2} \mid T_2) P(T_2)} \end{aligned}$$.

If the prevalence of the different types is equal, P($T_1$) = P($T_2$), then the priors are equal and selecting the type with the largest likelihood is equivalent to assigning the weights $w_{T}(d_{i,j})=\text {log}(P(D_{i,j}=d_{i,j} \mid T_k))$. Our goal is to estimate $P(D_{i,j} \mid T_k)$ for the decision-makers and different image types. If we allow for the prevalence to be different (which is the case for the test images), then we need to include the prior term for each lesion type. If $\pi _T$ is the prevalence of *T*, we have the following expression.

$$\begin{aligned} D_{i}= \underset{T \in \mathbb {T}}{\text {argmax}} \left[ \left( \sum _{d \in C_i}log(P(D_{i,j}=d_{i,j} \mid T_k)) \right) + \text {log}(\pi _T) \right] \end{aligned}$$

#### Equal weighting (LAW-E)

In the equal weighting algorithm, we use the mean training accuracy of the individual to estimate the probability of the different types. Suppose the accuracy of the participant $P_j$ is $a_j$. Suppose this individual sees a test image of type *T*. Based on the training images, the probability that they are correct is $a_j$. Hence, we estimate the probability that $d_{i,j}$ is *T* as $a_j$. The probability that $d_{i,j}$ is not *T* is $1-a_j$. For this algorithm, we assume that it is equally likely for the decision to be any of the other $\Vert \mathbb {T}\Vert -1$ types. Note that $\Vert \mathbb {T}\Vert$ represents the number of image types, which is 7 in our case. Hence, we estimate the probability of the decisions to be one of the other (non-selected) types as $(1-a_j)/(\Vert \mathbb {T}\Vert -1)$.

Hence, we weigh the decision for the selected type by $log(a_j)$ and for the types that were not selected by $log((1-a_j)/(\Vert \mathbb {T}\Vert -1))$:

$$\begin{aligned} w_{T}(d_{i,j})= {\left\{ \begin{array}{ll} log(a_j)\ \text {if}\ d_{i,j}=T\\ log((1-a_j)/(\Vert \mathbb {T}\Vert - 1)) \text {if} \ d_{i,j} \ne T \end{array}\right. } \end{aligned}$$

We note that if the training accuracy $a_j$ is 0 or 1, either the top term or the bottom term becomes undefined. To fix this, we include a threshold parameter $0< \tau < 1$. We constrain training accuracy to the range $\tau$ to $1-\tau$ by placing these as hard boundaries. That is, if the accuracy is lower than $\tau,$ then we replace it by $\tau$ or greater than $1-\tau$ and then we replace it by $1-\tau$. In the main paper, we set $\tau = 0.02$, and we vary $\tau$ in supplement to show that unless $\tau$ is large (above 0.1), it does not change the results.

#### Confusion-all weighting (LAW-CA)

In the confusion-all weighting algorithm, we incorporate information about the pattern of classification mistakes in training. For example, MEL and NV appear similar to each other and are often confused. For an NV image, one might respond MEL more often than AKIEC. Let $c_{d_{i,j},T}$ represent the probability when the true class is *T*, the selected class is $d_{i,j}$ in training. These weights are identical to the ones depicted in the top left panel of Fig. 2. For this algorithm, we calculate these values at the group level (see top panel of Fig. 2). While estimating these numbers at the group level allows us to have accurate estimates for each of the terms of the confusion matrix, it ignores the individual differences in training accuracy and response styles. We define the weights as follows:

$$\begin{aligned} w_{T}(d_{i,j})=log(c_{d_{i,j},T}) \end{aligned}$$

#### Confusion-individual user weighting (LAW-CI)

In the confusion-individual user weighting algorithm, we account for individual differences in the pattern of responses as illustrated in the bottom panels of Fig. 2. For instance, a participant might be biased toward selecting one image type versus another because of biases in their training data, response style, or prior knowledge. Let $c_{d_{i,j},T}$ represent the probability during training that when the true class is *T*, the selected class is $d_{i,j}$, for participant $P_j$ and training image *i*. We define the weights as follows.

$$\begin{aligned} w_{T}(d_{i,j})=log(c_{d_{i,j},T}) \end{aligned}$$

As mentioned in the previous section, we constrain these values to stay in the range $\tau$ to $1-\tau$. We conduct a sensitivity analysis in the supplement where we vary $\tau$ to show that as long as it is not too large (above 0.1), the results are similar.

While this algorithm accounts for the individual differences in training accuracy and response styles, these estimates might be noisy for each of the terms due to insufficient data. Hence, we estimate these weights for the 51 individuals who made 100 decisions or more on the training data, which constitutes $95.2\%$ of the train set. For the remaining individuals, we use the confusion matrix that was calculated at the group level. In the supplement, we restrict the data to the individuals who made 500 or more train decisions and the results are qualitatively the same.

We also include variants of these algorithms that account for the prevalence of different lesions. For the names of each of these algorithms, we use an additional *P* to indicate the use of the prevalence priors. That is, the variants of the algorithms that use priors are LAW-P-E, LAW-P-CA, and LAW-P-CI for LAW-E, LAW-CA, and LAW-CI, respectively. We estimate the prevalence of different lesions based on the training data as shown in Table 1.

### Switchboard analysis

In the section above, we described algorithms based on two main techniques—selection and weighting. It is possible to combine both selection and weighting into a single algorithm. Let $S_{i}$ be the subset of selected participants for the *i*th image and $w'$ be an accuracy weighting scheme. That is, if the individual is in the selected subset $S_i$, then the decision is weighted based on the weighting scheme $w'_T$.

$$\begin{aligned} w_{T}(d)= w'_{T} (d){\textbf {1}}_{S_i} \end{aligned}$$

In this paper, we conduct a full switchboard analysis where we investigate all of our different ways of selection and combine them with the different ways of accuracy weighting.

### Metrics

Different metrics capture different aspects of the performance (Hand, 2006, 2012). Depending on the real-world application, one might consider a different performance metric that needs to be maximized. Following Tschandl et al. (2019), we capture the performance of the crowd with four different metrics:

#### Metrics based on final decision

The first two metrics only look at the final decision of the algorithm.

- **Accuracy**: The first metric is the mean accuracy. This is the average probability that the crowd is correct. A response bias toward the classes with higher prevalence might increase the overall accuracy since it constitutes most of the test classes. Since our test dataset is imbalanced with one class, NV, having more images than the others, one might achieve a higher accuracy by performing well on NV but not on other classes. For example, a decision-maker that responds ‘NV’ on all images will have an accuracy of $60.1\%$ (equal to the prevalence of ‘NV’ in the test set) since they will get all of the ‘NV’ images correct and all other images incorrect.
- **Balanced accuracy (Mean sensitivity).** This is the mean sensitivity score for each class. The sensitivity is the fraction of the lesions of Type *T* that have correctly been identified (Grandini et al., 2020). Specifically, if $\text {TP}_T$ is the number of true positive cases of type *T* and $\text {FN}_T$ is the number of false negative cases for lesion type *T*, the sensitivity for type *T* is given by $\frac{\text {TP}_T}{\text {TP}_T+\text {FN}_T}$. The balanced accuracy is given by:
  $$\begin{aligned} \text {Balanced Accuracy} = \sum _{T \in \mathbb {T}}(\text {Sensitivity}_T)/|\mathbb {T}| = \sum _{T \in |\mathbb {T}|}\frac{\text {TP}_T}{\text {TP}_T+\text {FN}_T}/|\mathbb {T}| \end{aligned}$$
  The goal of the balanced accuracy metric is to give equal weight to decisions for all lesion types. For any given lesion class—$T_1$, one can achieve a perfect sensitivity score of 1 by always responding $T_1$. However, this impacts the sensitivity of all of the other classes. For instance, suppose a decision-maker responds ‘NV’ on all of their trials, they will never be wrong with the images of type ‘NV.’ Hence, they will have a perfect sensitivity of 1.0 for the lesion of type ‘NV.’ However, they will have a sensitivity of 0 on all of the other classes since they are not ‘NV.’ Hence, their mean sensitivity in this case will be $1/7=14.2\%$ while maintaining an accuracy of $60.1\%$. In some real-world cases, the performance on rare lesions might not be as important as the performance on the more common lesions. Here, one might focus on the accuracy metric. In cases where the rare lesions are equally important as the more common ones, one might want to focus on the balanced accuracy metric.

#### Metrics based on weights of different classes

In medicine, not all the mistakes are equal. Thus, we might adaptively apply different thresholds to either be cautious about making misses or false alarms. Suppose the outputs of the algorithm for the seven different lesion types are $(w_1, w_2,... w_7)$. Hence, instead of restricting ourselves only to the final decision, or the lesion with the maximum weight, we might use the weight given to each lesion class to evaluate each of the algorithms. For this purpose, we introduce metrics that measure the algorithm’s ability to trade-off between false alarms and misses. We introduce two measures of the area under the curve of the receiver operating characteristic (ROC-AUC).

- **Mean ROC-AUC:** The mean ROC-AUC is the mean of the 7 ROC-AUC values which is calculated based on a one vs. the rest classification for the 7 different lesion types. Each of the 7 weights helps in making a trade-off between false alarms and misses of the seven different classes. For a high mean ROC-AUC, each of these 7 terms needs to be informative about the trade-off. Hence, the mean ROC-AUC metric captures the ability to trade-off between false alarms and misses of all seven different classification decisions.
- **Malignant ROC-AUC:** One might be interested only in the binary classification of lesions as malignant versus not malignant. We group the lesion types into malignant types—AKIEC, BCC and MEL and non-malignant types—BKL, DF, NV, and VASC. We then calculate the ROC-AUC of the different algorithms. For this, the total weight given to the cancer types $w_{\text {cancer}} = w_{\text {AKIEC}}+w_{\text {BCC}}+w_{\text {MEL}}$ and total weight given to the non-cancerous types $w_{\text {non-cancer}}=w_{\text {BKL}}+w_{\text {DF}}+w_{\text {NV}}+w_{\text {VASC}}$ are important but the distribution within each of the sub-classes is not important. Hence, the malignant ROC-AUC metric captures the ability to trade-off between false alarms and misses between malignant and non-malignant classification decisions. Discriminating between the specific kind of malignancy and non-malignancy is not as important.

## Results

We present the results obtained from applying the modeling methods mentioned above.

### Simple voting

**Fig. 3:**
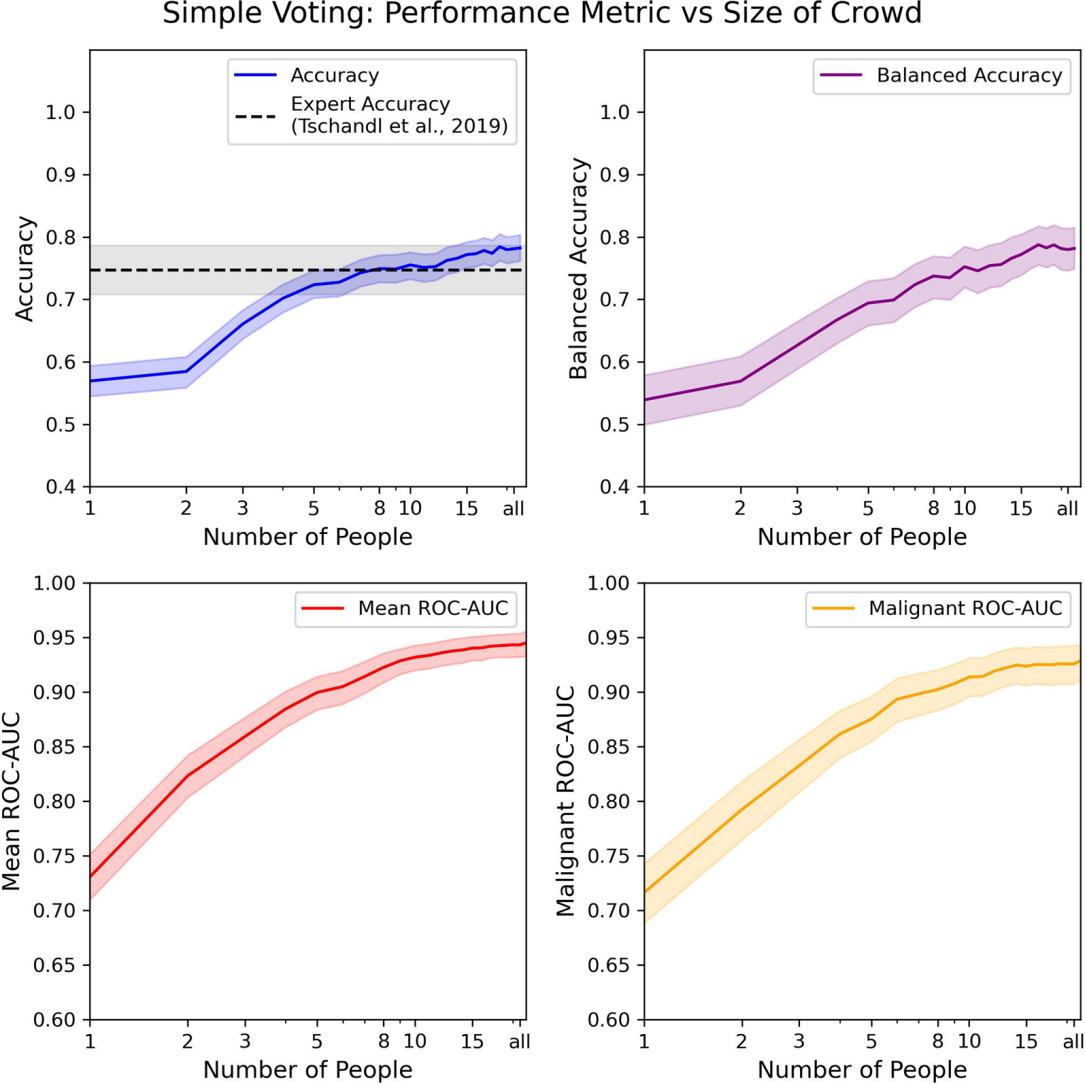
The performance of simple voting on the different metrics based on the size of the crowd. The left panel shows the accuracy and balanced accuracy metrics and the right panel shows the mean ROC-AUC and malignant ROC-AUC. The 95% bootstrapped confidence intervals are depicted as transparent bands around the line

We estimated the performance of simple voting for a crowd of varying sizes (*n*) to obtain a baseline. We estimated the performance of simple voting for a given *n* by randomly choosing decisions such that there were *n* decisions on every image. We used this subset to estimate the performance of the group of size *n*. As shown in Fig. 3, we observed that the performance improved across the different metrics as the group was made larger.

As shown in Fig. 3, when one person’s decision was considered, the accuracy was $56.9\%$. We note that this is slightly different from the mean accuracy reported on the test set since only one decision for every image was selected before making an estimate. Accuracy rose to $74.9\%$ when 8 decisions were used, matching the performance of a single dermatologist at $74.7\%$ (Tschandl et al., 2019). The crowd’s performance exceeded expert performance when all of the decisions were used by achieving an accuracy of $78.2\%$. The results similarly improved balanced accuracy when more decisions were aggregated. When only one decision was considered, it was $53.9\%$ which rose to $73.7\%$ with 8 decisions and $78.2\%$ when all of the decisions were aggregated.

The mean ROC-AUC also increased from 0.731 when one decision was considered to 0.922 with 8 decisions and 0.945 when all of the decisions were aggregated. The malignant and non-malignant ROC-AUC increased from 0.716 when one decision was considered to 0.902 with 8 decisions and to 0.928 when all of the decisions were used.

This shows that including more people dramatically improves performance across different metrics as in Duhaime et al. (2023). The high values for the ROC-AUC indicate that the crowd was not just able to classify images into the correct class but also had the ability to capture a measure of the uncertainty in the classification.

### Algorithms based on selection weighting

**Fig. 4:**
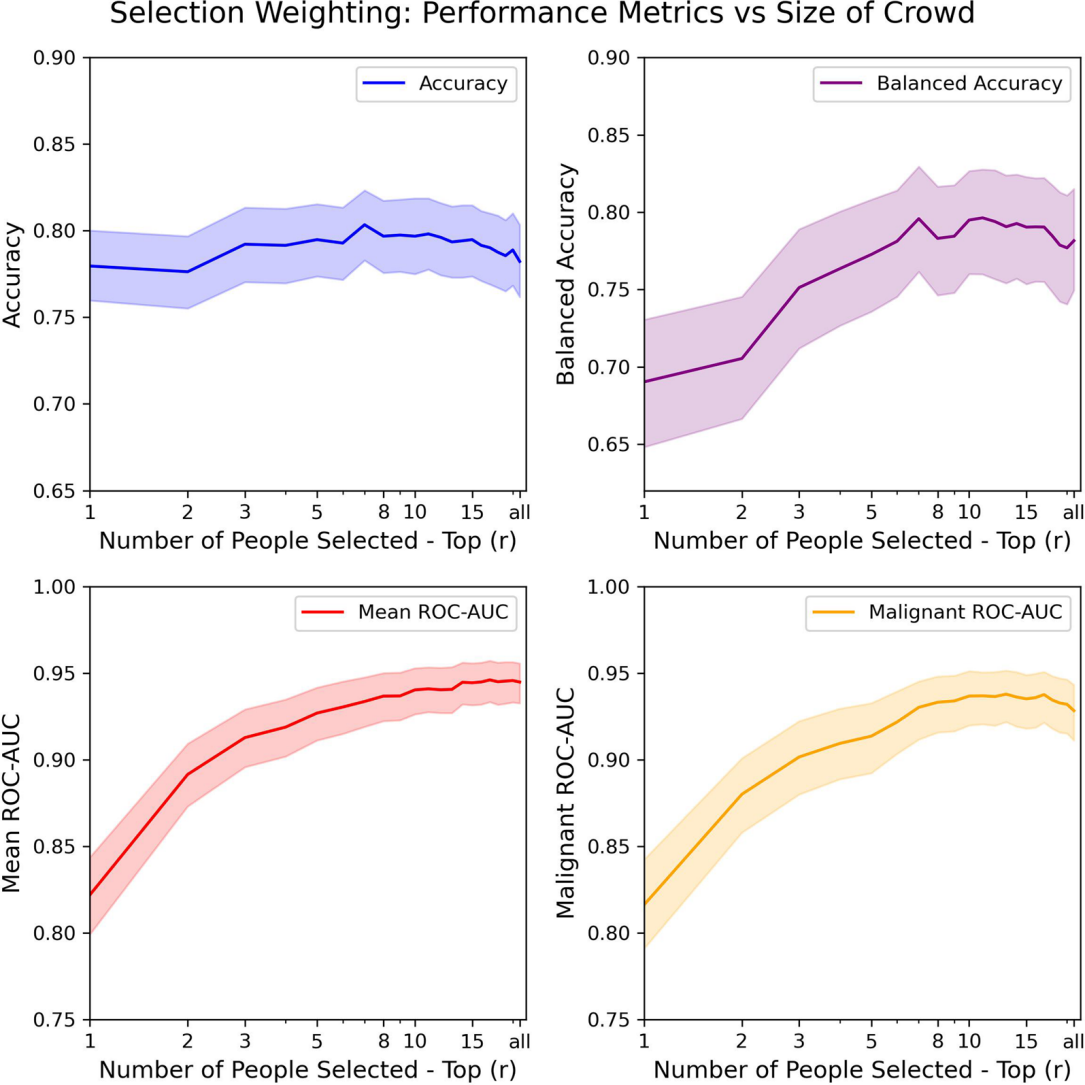
The results of the algorithms based on selecting the top individuals using their training performance. The 95% bootstrapped confidence intervals are depicted as transparent bands around the line

We tested the algorithms based on selection. We selected the top *r* individuals based on their accuracy on the training set and calculated their aggregated decisions on the test set. Our results are shown in Fig. 4.

We observed that the accuracy when the top individual was selected was $78.0\%$. The accuracy slightly rose to a maximum of $80.3\%$ when decisions from the top 7 people were aggregated. The accuracy slightly dropped to $78.4\%$ when decisions from 21 people were selected. Hence, we see that the accuracy of the crowd might improve slightly when individuals are selected based on their training accuracy.

The balanced accuracy for only selecting the top-performing individual was $69.0\%$ which is a lot lower than $78.2\%$ with simple voting. Hence, we see that when the top one or two individuals are selected, the balanced accuracy is lower than keeping everyone in the crowd. Balanced accuracy weights the performance on the rare classes as much as the performance for the more prevalent classes. We observed similar accuracy scores when retaining the entire crowd or when only the top 1 or 2 individuals were selected. However, we observed lower balanced accuracy scores when only the top 1 or 2 individuals were selected. This indicates a drop in the sensitivity of the rarer classes when only the top one or two individuals are selected to form the crowd. As more individuals were included, the balanced accuracy sharply rose to a maximum of $79.6\%$ when decisions from 11 individuals were aggregated. The balanced accuracy dropped slightly to $77.9\%$ when decisions from the top 21 people were selected. This indicates that there might be potential gains in balanced accuracy from selecting an optimal number of people.

The mean ROC-AUC and malignant ROC-AUC have a clear trend. We observed that the mean ROC-AUC was 0.822 when only the top individual was selected. The mean ROC-AUC consistently improved to 0.947 as more participants were included. Similarly, the malignant ROC-AUC started off at 0.784 and improved to a maximum of 0.938 when decisions from the top 13 people were aggregated and then gradually declined to 0.930 when decisions from 21 people were aggregated. Thus, we see that when more individuals are selected, one is better able to make trade-offs between the false alarms and misses compared to when only the top 1 or 2 performers form the crowd.

### Algorithms based on accuracy weighting

We tested the algorithms that depended on weighting the decisions based on the training accuracy of individuals. Specifically, as described in the modeling methods section, we tested the simple accuracy weighting algorithm (SAW) and weighting based on the log accuracy (LAW). When weighing by the log of the accuracy, we tested three different variants. The first one accounted for individual differences in accuracy but did not account for the patterns in the classification errors between the different types (LAW-E). The second one accounted for the pattern of errors between the different image types made at the group level but did not account for individual differences (LAW-CA). The third one accounted for the pattern of errors in the image type made at the individual level (LAW-CI).

**Fig. 5:**
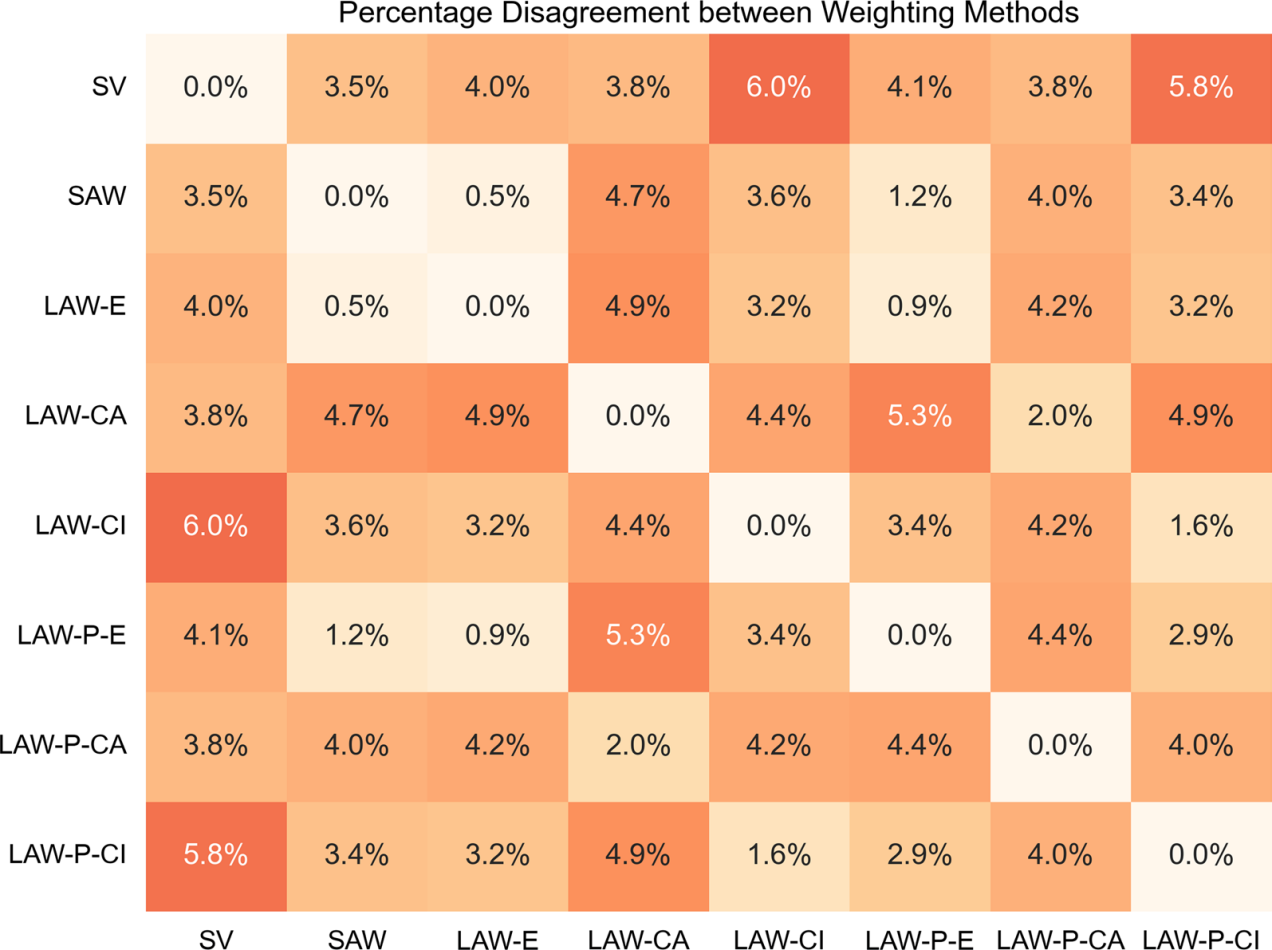
Inter-algorithm disagreement rate for accuracy weighting algorithms

First, we were interested in comparing how similar these algorithms were to each other. We calculated the inter-algorithm disagreement rate which was the fraction of the test images on which the decisions made by the different algorithms were different from each other (see Fig. 5). The SAW, LAW-E, and LAW-CA were pretty similar to SV (simple voting) and disagreed only on $3.5\%$, $4.0\%$ and $3.8\%$ of the cases. LAW-CI was maximally dissimilar to SV on $6.0\%$ of the cases.

Next, we compared the different accuracy weighting algorithms on the four key metrics as presented in Tables 3 and 4. We bootstrapped over the test set and compared two algorithms to each other on the sampled subset to estimate the uncertainty in the improvement. We present comparisons between each algorithm and simple voting in the main paper. The full pairwise comparison between all algorithms is presented in the supplement. On the accuracy metric, we observed that algorithms that accounted for individual differences, SAW and LAW-E (both had an accuracy of $79.6\%$), performed better than simple voting. As shown in Fig. 5, SAW and LAW-E were very similar to each other and disagreed only on $0.5\%$ of the decisions. Hence, weighting by logs or directly by the training accuracy gave very similar results in terms of the final decision. LAW-CI also performed slightly better than simple voting and had an accuracy of $79.0\%$ and balanced accuracy of $78.9\%$. We note that the difference in accuracy between LAW-CI and SV was only 0.8 percentage points, but the inter-algorithm disagreement rate was $6.0\%$. Thus, the difference between LAW-CI and SV was not just due to the improvement in the performance of LAW-CI. Rather, these two algorithms arrive at different decisions. Finally, LAW-CA, which did not account for individual differences, performed similar to simple voting.

**Table 3:** Comparison of the different accuracy weighting algorithms on accuracy and balanced accuracy

Abbreviation	Accuracy	Balanced accuracy
SV	78.2%	78.2%
SAW	79.6% [0.6%,2.3%]	79.9% [0.7%,3.0%]
LAW-E	79.6% [0.5%,2.4%]	79.9% [0.3%,3.3%]
LAW-CA	77.7% [−1.3%,0.2%]	77.7% [−2.1%,1.1%]
LAW-CI	79.0% [−0.2%,1.8%]	78.9% [−1.1%,2.6%]
LAW-P-E	79.8% [0.7%,2.6%]	79.5% [0.1%,2.9%]
LAW-P-CA	78.3% [−0.7%,0.8%]	76.9% [−2.7%,0.2%]
LAW-P-CI	79.4% [0.1%,2.2%]	78.7% [−1.3%,2.3%]

The square brackets show the bootstrapped 95% confidence intervals for the improvement as compared to simple voting

**Table 4:** Comparison of the different accuracy weighting algorithms

Abbreviation	Mean ROC-AUC	Malignant ROC-AUC
SV	0.945	0.928
SAW	0.949 [0.003,0.006]	0.935 [0.004,0.009]
LAW-E	0.953 [0.001,0.016]	0.935 [0.001,0.012]
LAW-CA	0.950 [0.000,0.012]	0.929 [−0.005,0.007]
LAW-CI	0.956 [0.005,0.019]	0.935 [−0.000,0.013]
LAW-P-E	0.954 [0.002,0.017]	0.936 [0.002,0.014]
LAW-P-CA	0.951 [0.001,0.013]	0.930 [−0.004,0.008]
LAW-P-CI	0.957 [0.006,0.020]	0.935 [0.001,0.014]

The square brackets contain the bootstrapped 95% confidence intervals for the improvement compared to simple voting

For the mean and malignant ROC-AUC score, the weighting algorithms performed better than simple voting. The mean ROC for simple voting was 0.945 and for SAW, LAW-E, LAW-CA and LAW-CI was 0.949, 0.953, 0.950 and 0.956, respectively. This indicates that the weighting algorithms might be able to provide a slightly more fine-grained ability to distinguish between the different skin lesion classes.

When we accounted for the prevalence of different image classes, using the modification described in the modeling methods, the decisions for LAW-E, LAW-CA and LAW-CI changed for $0.9\%$, $2.0\%$ and $1.6\%$ of the cases as shown in Fig. 5. Compared to similar algorithms that exclude the prevalence terms, the accuracy increased and the balanced accuracy decreased. Accounting for prevalence increases the response of image types with higher prevalence, which plays a larger role in the accuracy metrics. This decreases the correct identification of other lesion types, which plays a larger role in the balanced accuracy score. However, this effect is small since this extra term rarely overturns the decision of the entire crowd. The mean ROC-AUC and malignant ROC-AUC scores do not change too much indicating that the rate at which the trade-off between false alarms and misses of the different lesion types remains similar to the algorithms that do not account for prevalence.

### Switchboard analysis

**Fig. 6:**
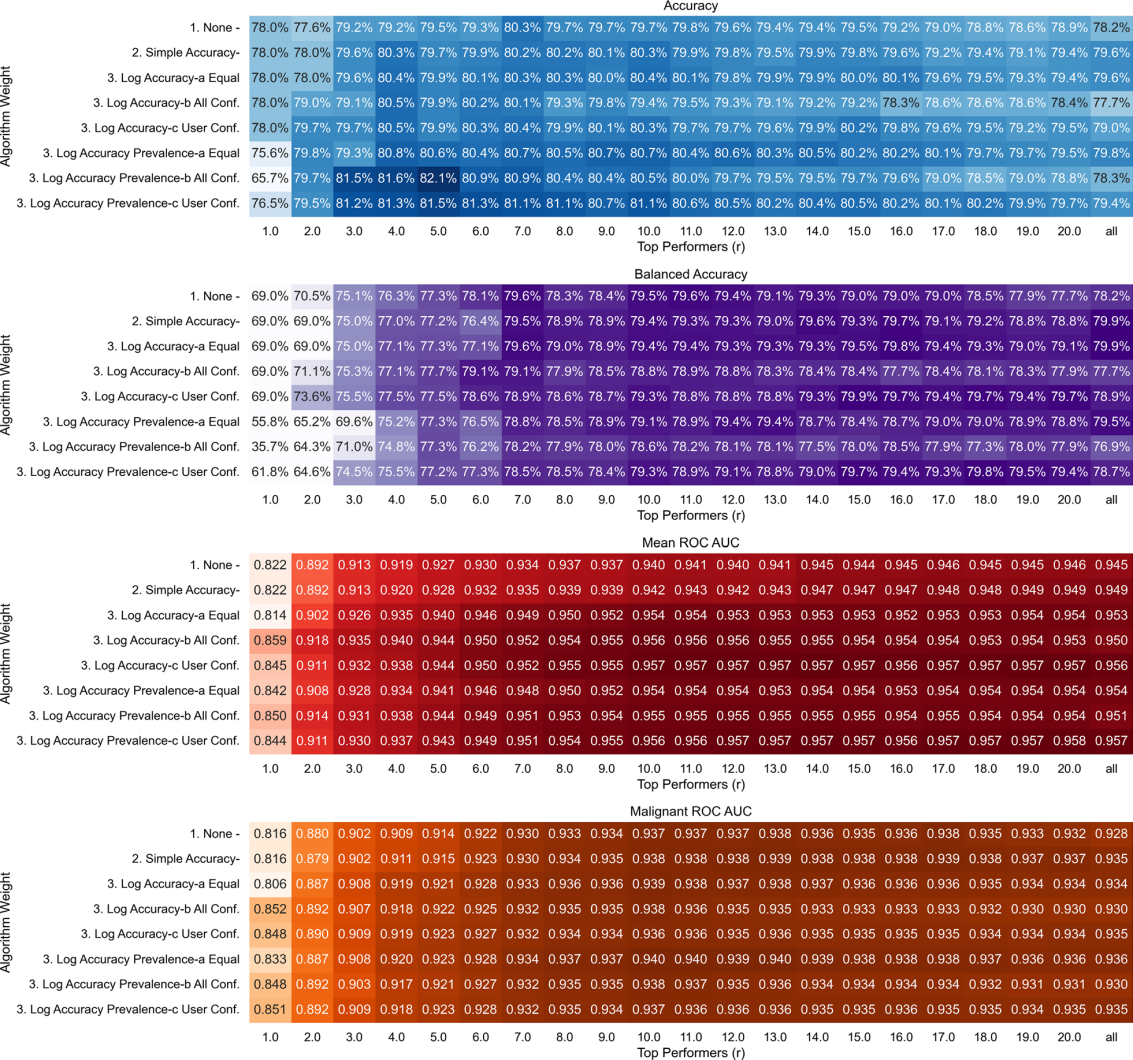
Switchboard analysis of all the different algorithms

We now conduct a full switchboard analysis to test the different hybrid algorithms. We present our results in Fig. 6. We make the following observations.

We observe that the accuracy ranges from 77.6% to $80.5\%$ and balanced accuracy ranges from 69.0% to $78.9\%$ depending on the aggregation algorithm when no prevalence information is used. The worst-performing algorithms, especially in the balanced accuracy metric, select only the top performers and exclude the rest. Regardless of the weighting method, the optimal number of top-performing individuals for our task was about 4–10. Across the different weighting methods, the best-performing algorithms were a combination of selection and accuracy weighting. Compared to algorithms based on selection weighting alone, for some accuracy weighting methods like SAW or LAW-E, retaining participants beyond the optimal number does not depreciate the performance notably across both metrics. This suggests that when weighted appropriately, one might not need the additional step of selection.

Similar to the algorithms based on selection alone, the mean ROC-AUC and malignant ROC-AUC continue to remain high when a large number of people are included in the crowd. The worst-performing algorithms were the ones that only used the top few performers. We observe that all the different accuracy weighting methods, especially the ones that used logarithmic weighting, had a higher mean ROC-AUC score compared to the ones dependent on selection alone. For the malignant ROC-AUC, we observed that one can achieve a high score even with selection weighting alone and no accuracy weighting when one uses between 10 and 13 of the top performers. However, we note that the accuracy-based weighting methods were robust to the inclusion of more participants beyond the optimal number compared to the ones that relied on selection alone, where the performance decreased slightly.

When we accounted for the prevalence as described in the modeling methods, we observed that the accuracy increased and the balanced accuracy decreased. As described in the previous section, this is due to the fact that the high prevalence classes play a larger role in accuracy than in balanced accuracy. When decisions from a small number of individuals are selected before aggregating, we observe that this difference is larger since the priors play a bigger role in the final decision. As more and more individuals are added, the algorithm becomes increasingly similar to the ones that ignore the priors and weight the decision of the crowd. However, we observe that the peak and decrease in performance suggests that the priors are being underweight compared to the decision when a large number of decisions are being aggregated. We observe that this modification does not change the algorithm’s ability to trade-off between false alarms and misses which is why the mean ROC-AUC and malignant ROC-AUC are similar to algorithms without the prevalence term.

## General discussion

In this paper, we used decisions obtained from an app-based interface to study the value of wisdom of crowds in medical image annotation (Duhaime et al., 2023). Given the wide range of accuracy and individual differences in patterns of errors, we compared different aggregation algorithms to produce a wisdom of the crowds in medical image decision-making that accounted for these differences. Overall, we observed that a simple voting aggregation strategy resulted in higher accuracy (78.2%) than that of a single dermatologist (74.7%), corroborating previous findings that wisdom of the crowds is an effective approach to labeling medical images (Hasan et al., 2023; Kurvers et al., 2016; Juni and Eckstein, 2017; Wolf et al., 2015; Duhaime et al., 2023). We also found further improvements in crowd performance by using more sophisticated strategies that selected top performers and weighted decisions by training accuracy. Specifically, the best algorithms improved performance over simple voting by around 3–4 percentage points for accuracy and around 1–2 percentage points for balanced accuracy metrics and mean ROC-AUC and malignant ROC-AUC by 0.01 points. We observed that while one might achieve high performance with selection weighting alone, using accuracy weighting in conjunction with selection makes the gains more robust beyond the optimal number of people, which might be crucial in practical applications when one does not know the optimal number of decisions. Although selecting a small crowd of top performers based on training images generally improved accuracy and balanced accuracy, we observed that selecting the top one or two performers hurt performance across different performance metrics. Finally, accounting for prevalence might help increase certain metrics such as accuracy but might hurt balanced accuracy, but not others such as the ROC-AUC, which is largely independent of the prevalence. Hence, we see that different algorithms might perform slightly better or worse based on the metric used to evaluate them. Depending on the specific use case, an individual might prefer one metric over another (Hand, 2006, 2012) and thereby select the aggregation algorithm that is best suited for that metric.

The results of our paper have important consequences for the labeling of medical images. First, using our approach, we obtained labels for a medical task at an accuracy that surpassed expert performance. Second, the data collection in our project took place over the span of 14 days, which is very quick for a dataset of this scale. If a single expert was to label the test set non-stop, assuming they take the median of 8.5 s per decision, they would take 215 h to label this dataset which amounts to more than 5 work weeks. Several such projects can take months to obtain high-quality labels (Cocos et al., 2017). Third, cost is a major factor in being able to determine the viability of such a project (Kentley et al., 2023; Ørting et al., 2020). By paying the crowd-sourced workers a total of $\$1,750$ in daily rewards over 14 days, Centaur Labs obtained 143,209 classification labels. This implies that the cost of an individual decision amounts to only $\$0.0122$ per decision. The cost of 8 decisions per image which matches expert performance is $\$0.097$, implying the dataset with 1511 images can be labeled for $\$146.57$. Accounting for a 50–50 test train split, the cost is less than $\$300$ to label the dataset with 1511 images. Fourth, when creating a new dataset in a different medical domain, one will need to identify specialized experts and create a new platform for recruitment and data collection for each application. In our case, the users signed up on the app for one task, could also be trained and deployed in another task, leading to a scalable solution. Hence, the app-based platform is accurate, fast, cost-effective and scalable to other medical tasks.

In our task, as we increased the number of individuals during the aggregation processes (that is, adding individuals randomly to the crowd and not based on training performance), all crowd-based performance metrics (i.e., accuracy, balanced accuracy, mean ROC-AUC, and malignant ROC-AUC) increased, showing a robust wisdom of the crowd effect (Duhaime et al., 2023). The increase in performance metrics was rapid at first but slowed down as more decisions were included, which is similar to the patterns in many tasks (Hasan et al., 2023; Hastie and Kameda, 2005; Galesic et al., 2018; Duhaime et al., 2023). Consistent with Hastie and Kameda (2005), we find that simple voting performed well in our task. The best-performing algorithm that did not use prevalence information, improved accuracy by 2.3 percentage points (i.e., LAW-CI with top-4 individuals) and balanced accuracy by 1.4 percentage points (No Weight-Top 11) compared to simple voting when all decisions were used. In high-stakes fields such as medicine, this improvement could lead to significantly superior downstream consequences especially when such a system is deployed at scale.

On metrics such as the mean ROC-AUC and malignant ROC-AUC, we observe that these metrics increase and continue to remain high even when the entire crowd is retained. This suggests that there is valuable information in the decisions of the low-performing individuals. This bolsters some of the wisdom of the crowd findings where novices, such as undergraduate psychology students, could learn to classify white blood cell images which when combined together exceeded expert performance (Hasan et al., 2023). Non-experts recruited in DiagnosUs with Centaur Labs showed that with a little training, crowds could identify complex lesion attributes (Kentley et al., 2023). This opens up the possibility of expanding the scope of citizen science projects (Cohn, 2008; Sullivan et al., 2014).

We observe that accuracy weighting improves performance across the different metrics, suggesting that it does well in our task, which is similar to previous research (Atanasov et al., 2017; Budescu and Chen, 2015; Wang et al., 2011, 2011b; Collins et al., 2023; Clemen, 1989). The log accuracy weighting does slightly better in the mean ROC-AUC and the malignant ROC-AUC, especially when aggregating decisions over a smaller number of people. Since these algorithms often create similar final responses, this rarely changes the final decision and hence is better reflected in the mean ROC-AUC scores and not in the accuracy or balanced accuracy. As described in the methods the mean ROC-AUC score depends not just on the final decision of the crowd but also on the ability to capture the uncertainty in the classification by trading off the false alarms and misses. This suggests that our Bayesian probabilistic treatment of the problem helps refine the final weights on the classes that were not selected, despite not changing the final decision. This is important since having a well-calibrated grasp on the uncertainty of the true label could help in the subsequent training of superior machine learning algorithms (Peterson et al., 2019; Schmarje et al., 2022; Uma et al., 2021; Collins et al., 2022).

Algorithms based on selection alone fared well when the optimal number of people were selected and did not improve much more when reweighted by training accuracy. This is partially because the dispersion in performance in the selected subset is lower than that of the group, reducing the need for weighting. Unlike the algorithms based on selection alone, we see that with SAW, LAW-E and LAW-CI, the decrease in performance is only slight for accuracy and that there is no real decrease in the ROC metrics. This is unlike LAW-CA which does not account for individual differences. This suggests that using accuracy along with selection can make the wisdom of the crowd algorithm more robust.

Algorithms that took the prevalence information into account improved the accuracy. Thus, accounting for the task environment helped improve the accuracy by boosting the weight of the more common classes. This is important since the prevalence of different classes is rarely equal in medical tasks and can result in decision-making biases (Wolfe et al., 2005; Trueblood et al., 2021). On the training data, the different lesions were randomized such that they were presented equally often. However, the test data had an unequal prevalence leading to certain decision-making biases as compared to the training set. Thus, intelligent aggregation algorithms should be able to take into account the task environment and related decision-making biases while keeping in mind the metric that needs to be optimized during the aggregation process (Galesic et al., 2018; Broomell and Davis-Stober, 2023).

For effective deployment of AI algorithms in the real world, it is important for the algorithms to be trusted by the individuals that use them (Glikson and Woolley, 2020). While the different wisdom of the algorithms have similar accuracy, the disagreement on their final decisions can be quite large. Thus, downstream AI trained based on this data will probably make different kinds of errors based on the training data. The kinds of errors made by an AI algorithm have important consequences for trust and continued reliance. When humans see algorithms err erroneously, they exhibit algorithm aversion, where they trust and use the algorithm less despite its overall accuracy (Dietvorst et al., 2015; Burton et al., 2020). For medical AI, it not just important to use procedures that lead to high accuracy but also to keep in mind the trust and utilization of algorithms for which some wisdom of the crowd algorithms might be better than others.

Finally, labels obtained using the wisdom of the crowd approach capture the human perceptual uncertainty in the classification. This has important consequences for downstream machine learning applications (Peterson et al., 2019; Schmarje et al., 2022; Uma et al., 2021; Collins et al., 2022). First, these uncertain labels allow machine learning algorithms to learn with fewer labels (Collins et al., 2022). Second, algorithms that were trained using labels that capture human uncertainty generalize better and are resistant to adversarial attacks (Peterson et al., 2019). Third, when algorithms are trained on discrete labels, they output overconfident scores (Schmarje et al., 2022). Finally, in medical situations where the real world is uncertain and ambiguous, capturing this uncertainty could be advantageous for human-AI collaborative decision-making. Developing algorithms for such applications is an active area of research (Uma et al., 2021; Schmarje et al., 2022).

### Future directions

A key question is whether the algorithms based on the probabilistic approach (i.e., log accuracy weighting) are the optimal choice. This depends on the accuracy of our estimates and the strength of the assumptions. We see in the supplement that even when restricting the analyses to individuals with many training responses, the results are similar, suggesting that better estimates of the quantities may not improve the results. Furthermore, whether our approach is optimal or not is also influenced by how we have modeled the independence assumption.

The assumption of independence has important theoretical implications since it has been shown to moderate the effectiveness of the wisdom of the crowds (Davis-Stober et al., 2014; Galesic et al., 2018; Mannes et al., 2014; Surowiecki, 2005; Clemen, 1989; Wilson and Farrow, 2018). Since individuals might use similar cues or have similar cognitive or perceptual biases, their decisions might be correlated (Galesic et al., 2018; Mannes et al., 2014; Wilson and Farrow, 2018). Modeling this correlation is a notoriously difficult problem (Clemen, 1989; Wilson and Farrow, 2018) and might require a large set of common images on which the same decision-makers have made decisions, unlike our set where every individual has made decisions on different images. It might be possible to parameterize inter-rater correlations to further improve aggregated decisions (Soule et al., 2023; Wilson and Farrow, 2018).

The approach adopted in this paper was to use a switchboard analysis where we tested several different ideas that are relevant to our question (Zhao et al., 2022; Turner et al., 2018). Other weighting approaches have weighted individuals based on their contribution by comparing the group performance with and without a given individual (Budescu and Chen, 2015; Chen et al., 2016). Using a optimization approach, one could find the best-performing algorithm by parameterizing different weight functions and maximizing the performance (Peterson et al., 2021). For instance, the ideal weight function could combine both selection and accuracy weighting in one function. Collins et al. (2023) proposed the use of a sigmoid weight function with a slope and inflection point, where if decisions are sufficiently far below the inflection point, they are down-weighted and effectively removed from the aggregate decision. The best-performing algorithm can be found by maximizing the metric of choice by varying the parameters. Future work can implement different approaches to find the optimal weights.

The reason we chose the switchboard analysis instead of the optimization approach was three-fold. One, our primary interest was to compare different wisdom of the crowd algorithms to understand overall trends. Using a switchboard analysis, we could ‘lay out’ all the algorithms we tested and look for systematic patterns. It is not easy to visualize results when one has three or more parameters. Second, the training set had a large variation in the number of decisions that were made on each image (IQR: 1–13 decision per image), making it difficult to use our training data for parameter estimation. Thus, one would need to fit the test data, but such an approach might overfit the testing data. One would need to create a validation set that is distinct from the test set so that these parameters can be found. Third, in terms of real-world crowdsourcing applications, it is often the case that the training set (often called ‘Gold Standards’) is small and the unlabeled image set that needs crowdsourcing (equivalent to the test set in our paper) is large. Of course, if one already has a large validated set, then our application may be irrelevant since it might be directly used to train a machine learning algorithm.

To further reduce the cost of labeling medical data, one might develop ‘online’ algorithms, which intelligently select the image that requires labeling. We do not need to keep collecting decisions on easy images and could instead spend more resources on hard images. If for instance, one observes consensus between the first few decision-makers on a given image, then it may not be necessary to collect a lot of decisions on that image since it is probably an easy image (Kurvers et al., 2019). On the other hand, for a difficult image, one might need to gather a lot of decisions to determine the true class. This could help further optimize resources and reduce the cost of data collection.

Further, the compensation framework could heavily impact the number and quality of decisions. In our task, the compensation framework predominantly favored top achievers, motivating their engagement with the app and subsequent image labeling. We also observed that the median number of daily responses per participant was around 100, which was the minimum number of decisions required to enter the tournament. It is also noteworthy that some individuals provided a lot more decisions than what was required to win the tournament, and provided a substantial portion of the responses. Future studies could look at alternative ways of incentivizing participation in the app, with the aim of improving engagement with the app and crowd-based performance metrics.

The gains from combining decisions from different sources are not limited only to aggregating human decisions. A similar framework for combining decisions from different machine algorithms has been developed (Kuncheva, 2014; Kuncheva and Rodriguez, 2014). More recently, ensemble approaches have been useful when combining decisions from neural networks in medical decision-making (Perez et al., 2019; Mahbod et al., 2020). Since humans and machines are susceptible to different biases (Steyvers et al., 2022; Tschandl et al., 2020), one might obtain additional gains by combining decisions from humans and machines.

### Constraints on generality

The field of medical decision-making has many different kinds of tasks. The example that we studied in this paper was complex classification (Kurvers et al., 2016; Hasan et al., 2023). However, one might also have a visual search task where one is looking for abnormalities in mammography for signs of cancer (Drew et al., 2013). Additionally, tasks might vary in format. For instance, in an image segmentation task, individuals highlight the lesion portion of the skin (Codella et al., 2019) or the task might be open-ended with different responses (Kurvers et al., 2023). Each of these tasks engages different cognitive processes and has a different pattern of errors. It is unclear to what extent our results might generalize across these different tasks.

When designing crowdsourcing tasks for medical data annotation, the efficacy of different algorithms might depend on task features such as the number of training cases and the spread in performance. For instance, we simulated the case where one has fewer training samples to calculate individual-level factors and present them in the supplement. We find that when one has fewer training cases (1–5 decisions per person), the simple accuracy weighting is worse than simple voting. As one has increasingly accurate estimates of accuracy (upwards of 20 decisions per person), the performance matches and starts exceeds simple voting. For more complicated algorithms like LAW-CI, one needs even more samples (upwards of 500 decisions per person), or else its performance is worse than simple voting. This is a reflection of the bias-variance trade-off (Brighton and Gigerenzer, 2015; Geurts, 2010), where simpler models lack the flexibility to account for patterns in the training data, leading to sub-optimal weights for decision aggregation. In contrast, more complex models might be over-sensitive to patterns in the training data, leading to inaccurate weights that hurt the crowd performance. Thus, in data-sparse environments, one might consider using simpler models and more complex algorithms in the data-rich environments. Further, for cases where the training data is sparse, one might develop and test machine learning algorithms that are not oversensitive to patterns in the training data (Williams, 1995; Moradi et al., 2020). This is similar to the results from the simulations run for ensemble studies with machine learning algorithms, where algorithms with more parameters need more training data before being included in the ensemble (Kuncheva, 2014; Kuncheva and Rodriguez, 2014). Further, if participants are recruited from similar sources with similar levels of skill, one might not gain by accounting for individual differences in performance. Thus, our results can be interpreted in the context of task features such as having a large number of training samples and recruiting a diverse set of individuals from an app-based platform.

The task that we study is a multiclass classification problem in which individuals provide discrete responses. It is unclear whether these results will generalize to other response formats. When individuals provide discrete categorical decisions, one outlier vote will not impact the decision of the entire crowd unless the crowd is split evenly across all possible responses. However, if one is interested in creating a dataset with well-calibrated confidence or probability judgments that use a continuous response scale, even a few outliers can strongly impact the final answer of the crowd (Budescu and Chen, 2015; Collins et al., 2023; Hasan et al., 2023; Litvinova et al., 2022). One might make substantial gains in calibration if these outlier values are removed before aggregation (Collins et al., 2023).

### Conclusion

In conclusion, we observe that simple voting performs well in our task despite the dispersion in individual performance. On a range of metrics, we observe that the best-performing algorithms both select the top performers and weigh them by their training accuracy. Taking into account the task environment, by incorporating the prevalence rates of different images, further improves the accuracy. We also observe that wisdom of the crowd approaches perform well on ROC-AUC scores, which is essential to developing algorithms that account for the uncertainty in classification. Overall, we observe that an app-based platform can be used to obtain accurate, cost-effective, fast, and scalable labels for medical image datasets.

### Supplementary Information

Supplementary material 1.

## Data Availability

Data will be made available upon reasonable request. The code is available on OSF—https://osf.io/c37mf
