# Supplementary material for: Boosting wisdom of the crowd for medical image annotation using training performance and task features
Source: Cogn Res Princ Implic. 2024 May 20;9:31. doi: 10.1186/s41235-024-00558-6 (PMC11102897; doi:10.1186/s41235-024-00558-6)
Supplement: Supplementary file 1 — Supplementary material 1. [file 41235_2024_558_MOESM1_ESM.pdf]

Boosting Wisdom of the Crowd for Medical Image Annotation Using Training Performance and Task Features

Author

[1] Department of Psychological and Brain Sciences, Indiana University

[2] Cognitive Science Program, Indiana University

[3] Centaur Labs

Corresponding Authors:

Eeshan Hasan

Department of Psychological and Brain Sciences, Indiana University

1101, E. 10th St. Bloomington, IN, USA 47405-7007

Email: eehasan@iu.edu

Jennifer S. Trueblood

Department of Psychological and Brain Sciences, Indiana University

1101, E. 10th St. Bloomington, IN, USA 47405-7007

Email: jstruebl@iu.edu

Boosting Wisdom of the Crowd for Medical Image Annotation Using Training Performance and Task Features

Demographics

Country Wise Distribution

In the main paper, we gave the different continents that participants came from. We now present the countries that they came from in Table 1.

Country	No. of People	Percentage
United States	90	28.57%
Namibia	54	17.14%
Ghana	24	7.62%
Mexico	20	6.35%
Romania	14	4.44%
Armenia	9	2.86%
Philippines	7	2.22%
Canada	6	1.9%
Georgia	5	1.59%
Poland	5	1.59%
United Kingdom	5	1.59%
Egypt	5	1.59%
Vietnam	5	1.59%
Turkey	4	1.27%
Pakistan	3	0.95%
Lebanon	3	0.95%
Bulgaria	3	0.95%
Brazil	3	0.95%
India	2	0.63%
Latvia	2	0.63%
Italy	2	0.63%
Russia	2	0.63%
Croatia	2	0.63%
Jordan	2	0.63%
South Africa	1	0.32%
Morocco	1	0.32%
Jamaica	1	0.32%
Colombia	1	0.32%
Iraq	1	0.32%
Israel	1	0.32%
Palestine, State of	1	0.32%
Germany	1	0.32%
Belgium	1	0.32%
Azerbaijan	1	0.32%
Sri Lanka	1	0.32%
Korea, Republic of	1	0.32%
Chile	1	0.32%
Bahamas	1	0.32%
Nepal	1	0.32%
Nigeria	1	0.32%
Algeria	1	0.32%
Netherlands	1	0.32%
China	1	0.32%
Argentina	1	0.32%
France	1	0.32%
Myanmar	1	0.32%
Thailand	1	0.32%

Table 1
The countries that the different participants came from.

Accuracy vs Occupation and Experience

We test if accuracy is related to occupation and years of dermatology experience. We present our results in Figure 1.

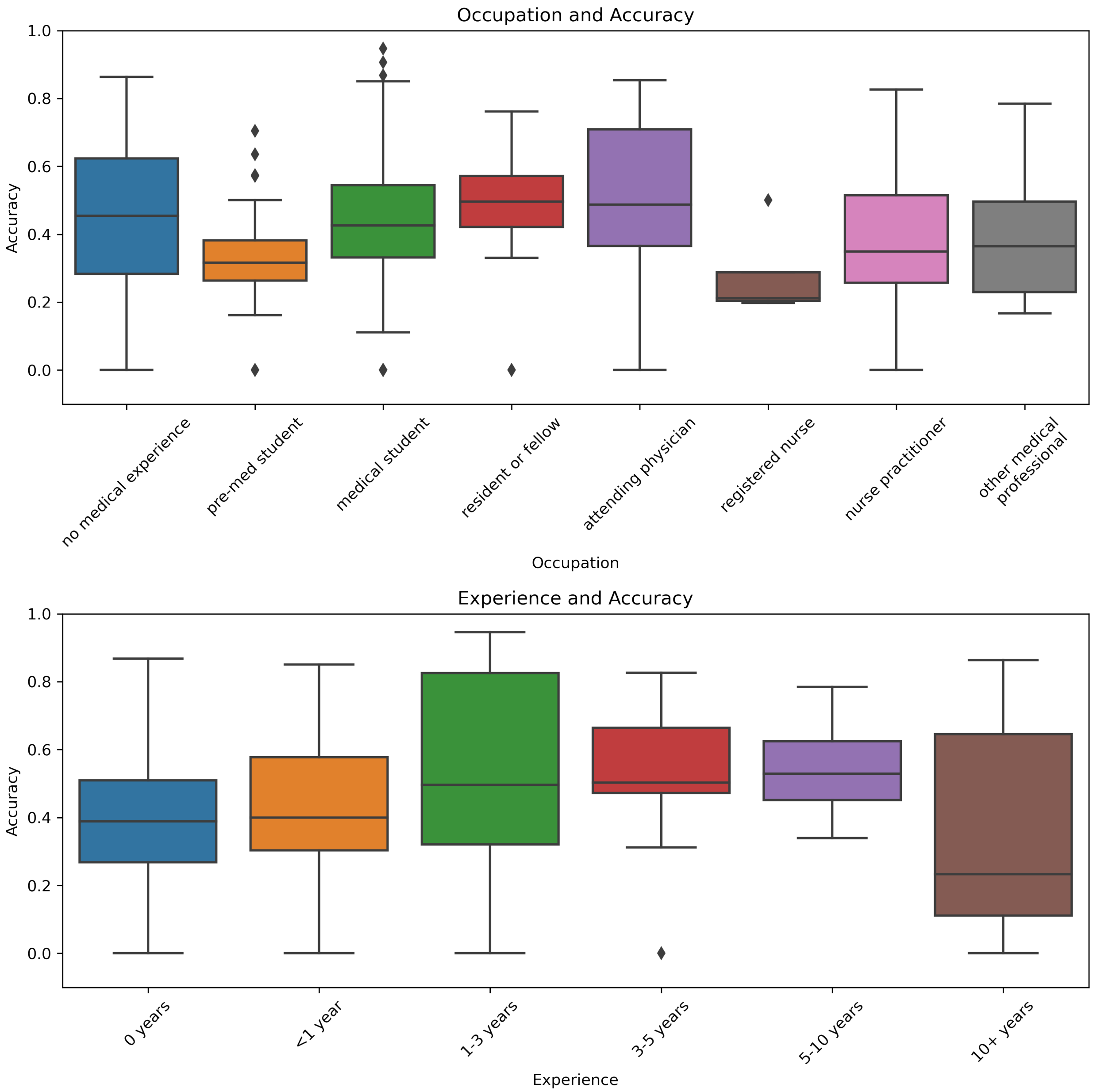

Figure 1

The distribution of accuracy based on occupation and years of dermatology experience

Daily Responses

Participants were given prizes based on their performance on a daily basis. To win the competition, the participant needed to make at least 100 decisions. We present the distribution of responses made per participant in Table 2. We observe that the median number of decisions on most days was around 100.

Table 2

Statistics regarding the distribution of the number of decisions contributed per participant on every day.

	No. of Participants	mean	min	25%	50%	75%	max
Day 1	102.0	124.1	1.0	53.5	100.0	120.0	723.0
Day 2	90.0	85.2	1.0	18.8	91.0	114.2	523.0
Day 3	80.0	107.0	1.0	27.0	93.5	108.5	1495.0
Day 4	86.0	131.7	1.0	51.2	101.0	123.0	1458.0
Day 5	88.0	126.1	1.0	44.0	100.0	133.2	1752.0
Day 6	102.0	136.5	1.0	27.8	100.0	139.5	1430.0
Day 7	82.0	125.7	1.0	31.0	100.0	120.0	696.0
Day 8	68.0	149.3	1.0	26.8	98.0	107.2	2450.0
Day 9	64.0	105.3	1.0	24.2	99.0	108.5	718.0
Day 10	61.0	132.0	2.0	26.0	100.0	132.0	1226.0
Day 11	65.0	118.0	1.0	26.0	99.0	130.0	1341.0
Day 12	73.0	172.9	2.0	44.0	100.0	136.0	1452.0
Day 13	72.0	139.4	1.0	33.0	100.0	108.0	2255.0
Day 14	65.0	190.6	2.0	31.0	100.0	120.0	4218.0
Mean	78.4	131.7	1.2	33.2	98.7	121.4	1552.6

Learning

In this section, we address the question of whether the training accuracy of participants changes during the task. We present our results in Figure 2. We plot the rolling average accuracy and a linear model fit to the accuracy of the participants who made the most decisions during the task. We observe that while some participants continue to improve at the task, others do not.

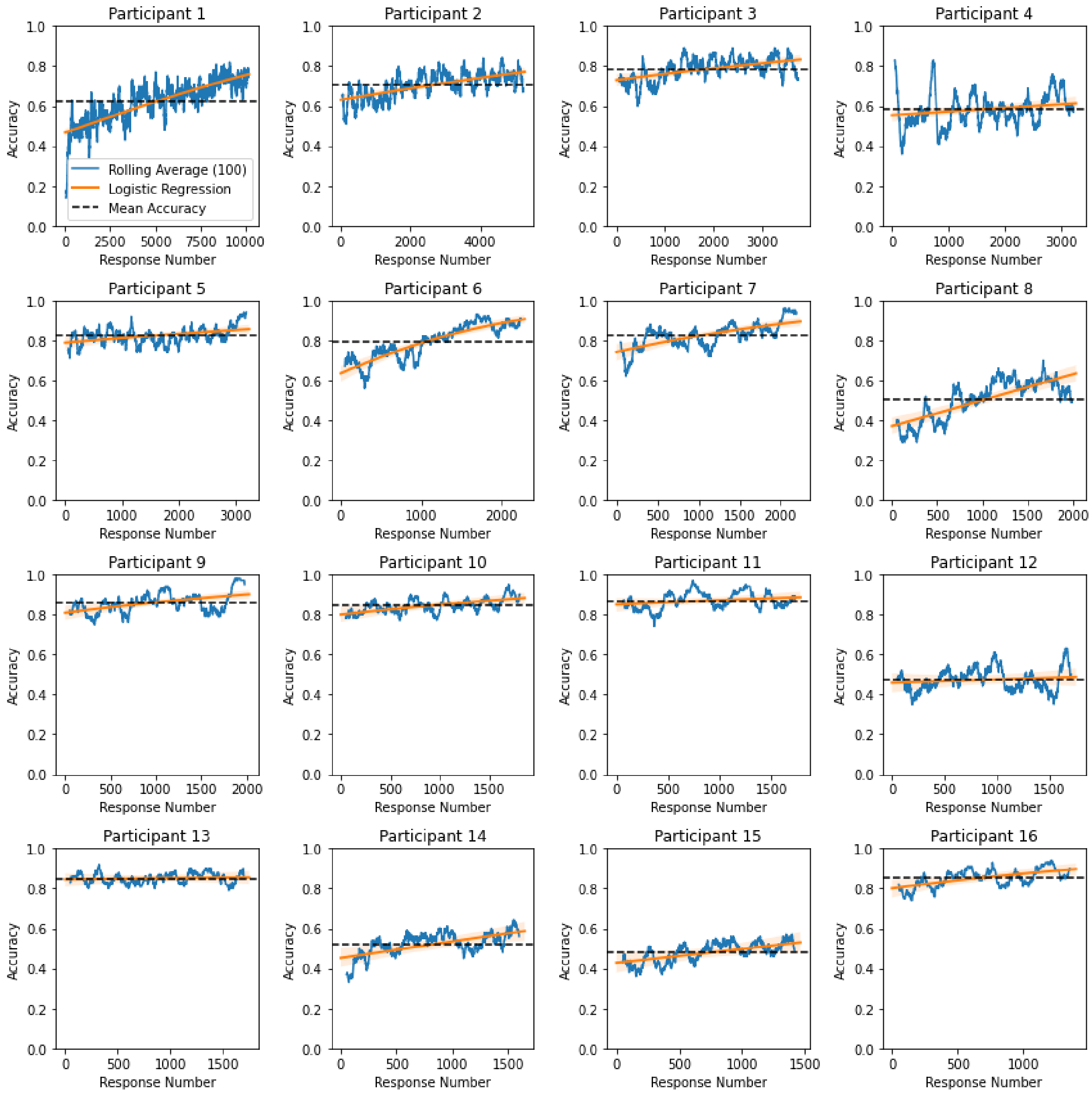

Figure 2

Rolling average training accuracy and a linear model fit to the accuracy of the participants who made the most decisions during the task.

Individual Differences in Confusion Matrices

In this section, we address the question of individual differences in response patterns. We plot the confusion matrices for the individuals that have made the most decisions on the training set.

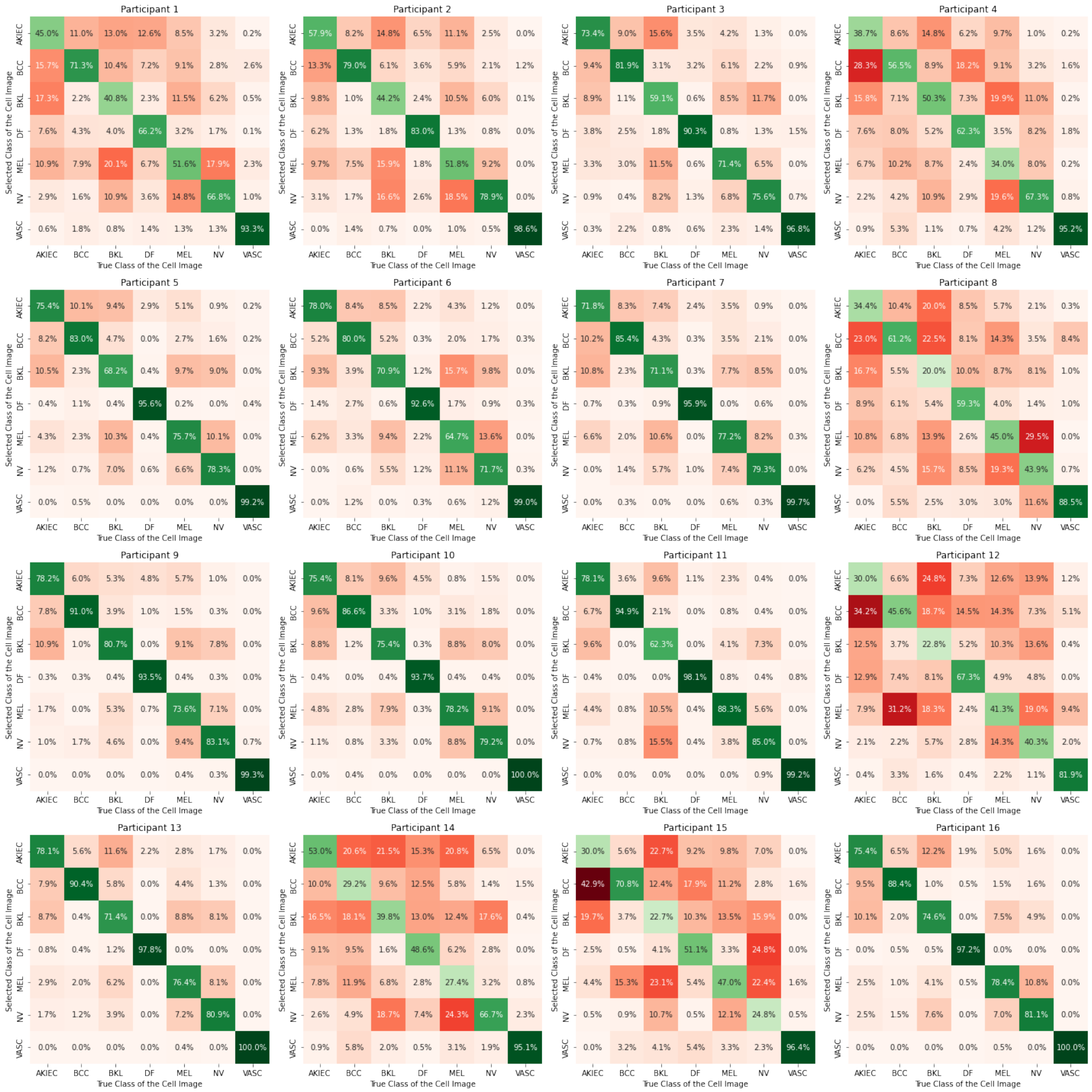

Figure 3

The confusion matrices based on the training data for the individuals with the most decisions.

Estimating Uncertainty

We estimated the uncertainty in each of the four metrics by bootstrapping over test set images. Specifically, we re-sampled images from the test set with replacement while ensuring that the number of images from each class was the same as the original test set. We applied the two algorithms that we were interested in comparing on this re-sampled subset. For each sample, we

estimated the 4 metrics and calculated the improvement of one algorithm relative to another. After applying this procedure 1000 times, we calculated the 2.5th percentile as the lower bound of the estimate and 97.5th percentile as the upper bound of the estimate of the improvement. We present our results in Figures 4, 5, 6 and 7.

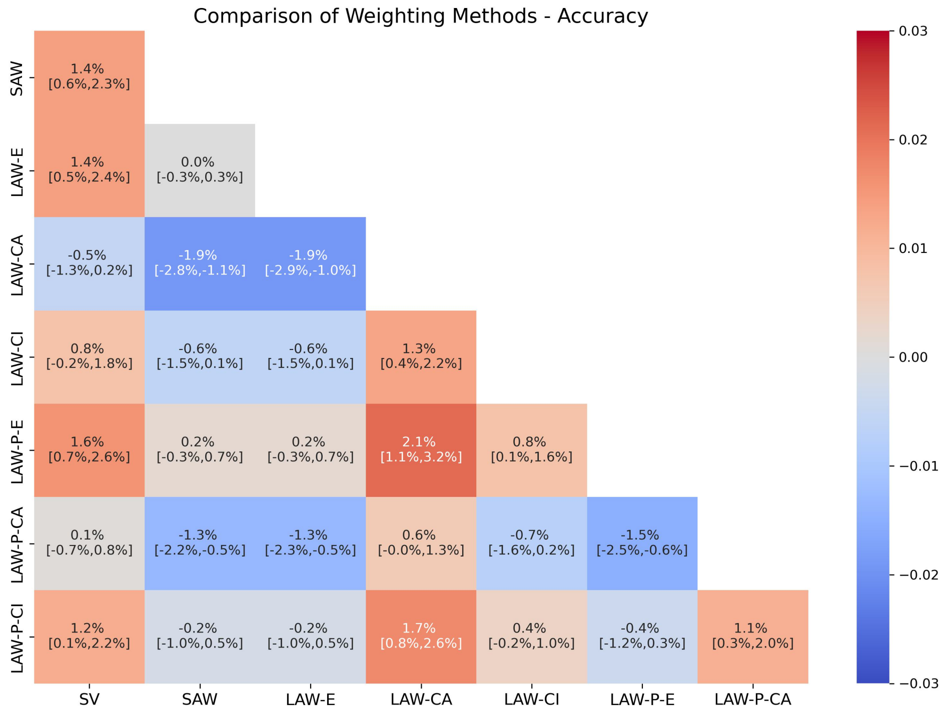

Figure 4

Each cell depicts the improvement in accuracy of the algorithm on the left compared to the algorithm on the bottom. A positive number indicates that the algorithm on the left has superior performance compared to the algorithm on the bottom.

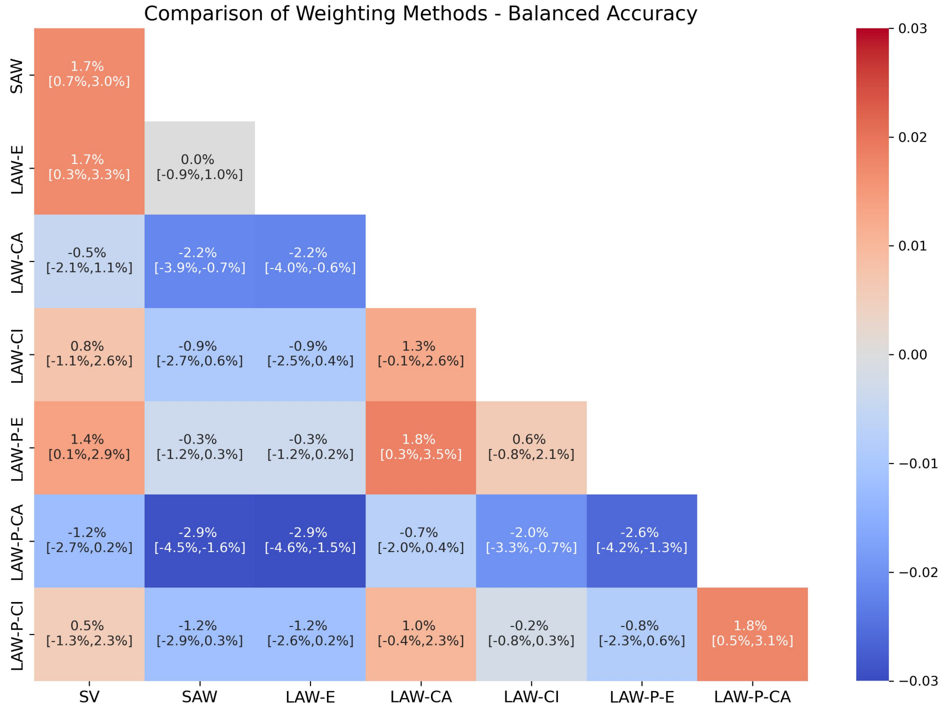

Figure 5

Each cell depicts the improvement in balanced accuracy of the algorithm on the left compared to the algorithm on the bottom. A positive number indicates that the algorithm on the left has superior performance compared to the algorithm on the bottom.

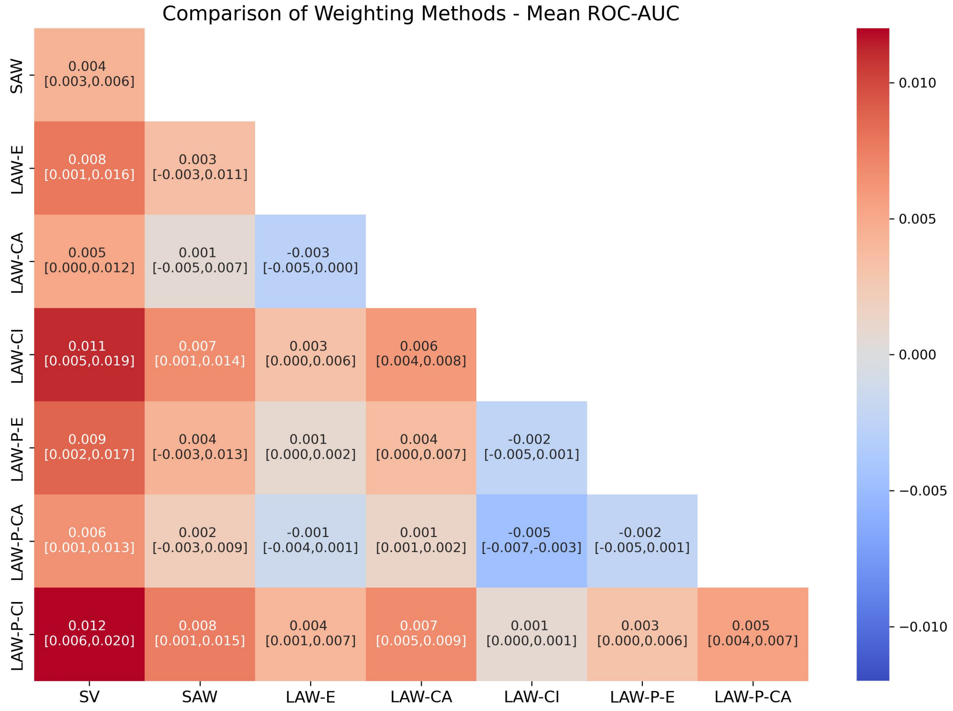

Figure 6

Each cell depicts the improvement in mean ROC-AUC of the algorithm on the left compared to the algorithm on the bottom. A positive number indicates that the algorithm on the left has superior performance compared to the algorithm on the bottom.

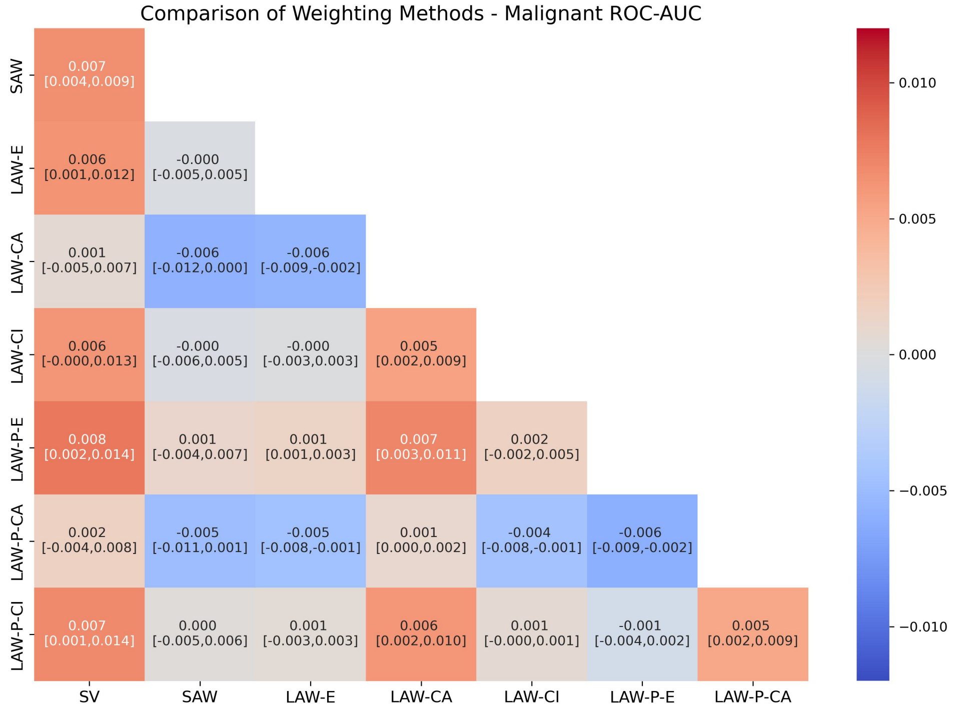

Figure 7

Each cell depicts the improvement in malignant ROC-AUC of the algorithm on the left compared to the algorithm on the bottom. A positive number indicates that the algorithm on the left has superior performance compared to the algorithm on the bottom.

Noisy Estimates of Accuracy

In our experimental set-up, we had a median of 101 training decisions from each individual to estimate the individual parameters. In other set-ups, one may have fewer training responses per individual. To test if our results are robust to different set-ups, we estimated the individual parameters using a maximum of 1, 5, 10, 20, 50, 100 and 500 training decisions per individual. We test each algorithm using these more noisy individual estimates. We present our results in Figures 8, 9, 10 and 11.

We observed that all of the algorithms perform worse than simple voting when very few training decisions are used to estimate individual parameters. We also observed that for more sophisticated algorithms that rely on individual patterns of responses (eg. LAW-CI), more training

decisions are required to estimate individual parameters compared to simpler algorithms that rely on estimates of individual accuracy (eg. SAW, LAW-E).

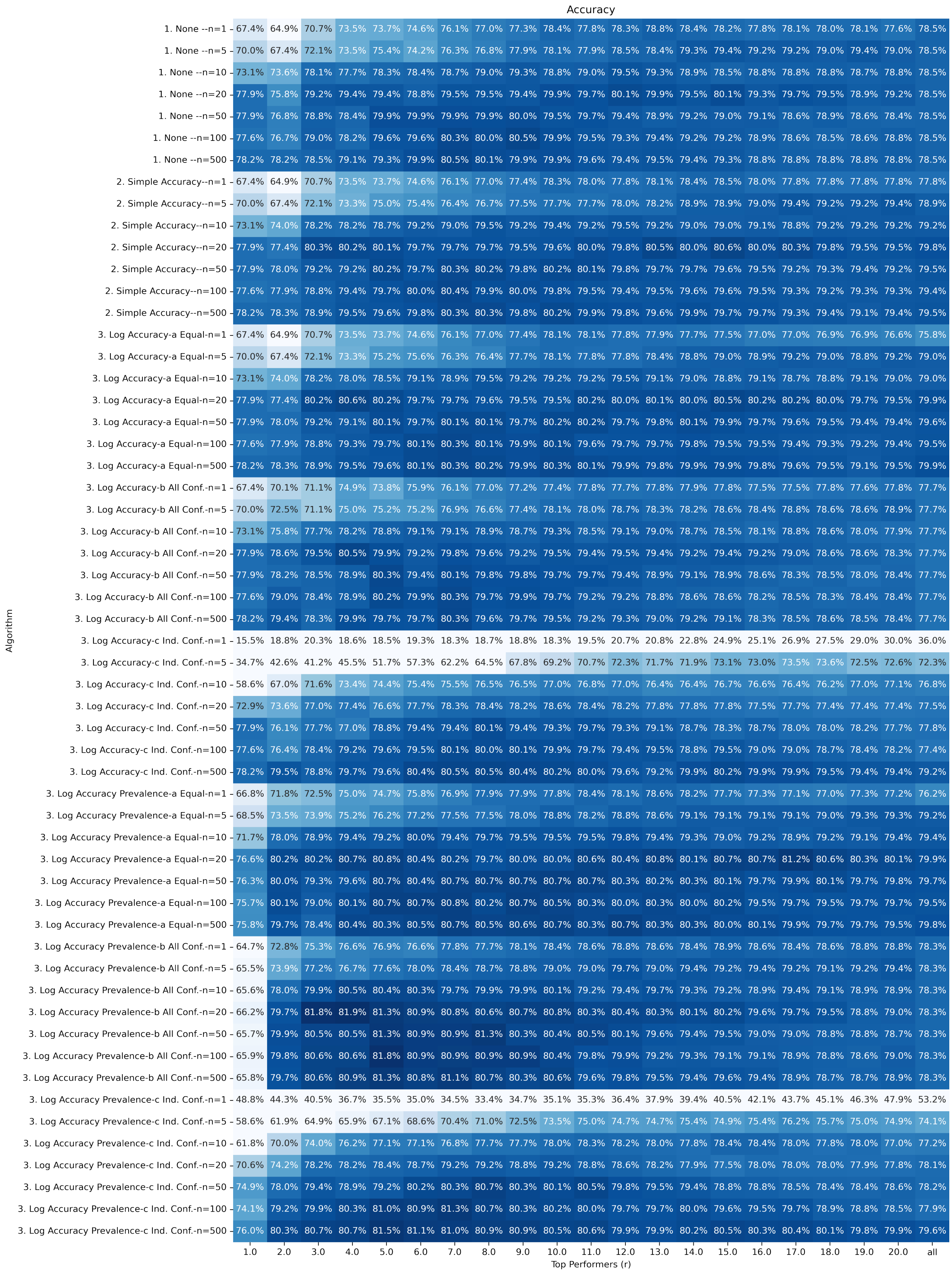

Figure 8

Estimating the accuracy of different algorithms while varying the number of training decisions (n) used to estimate individual parameters.

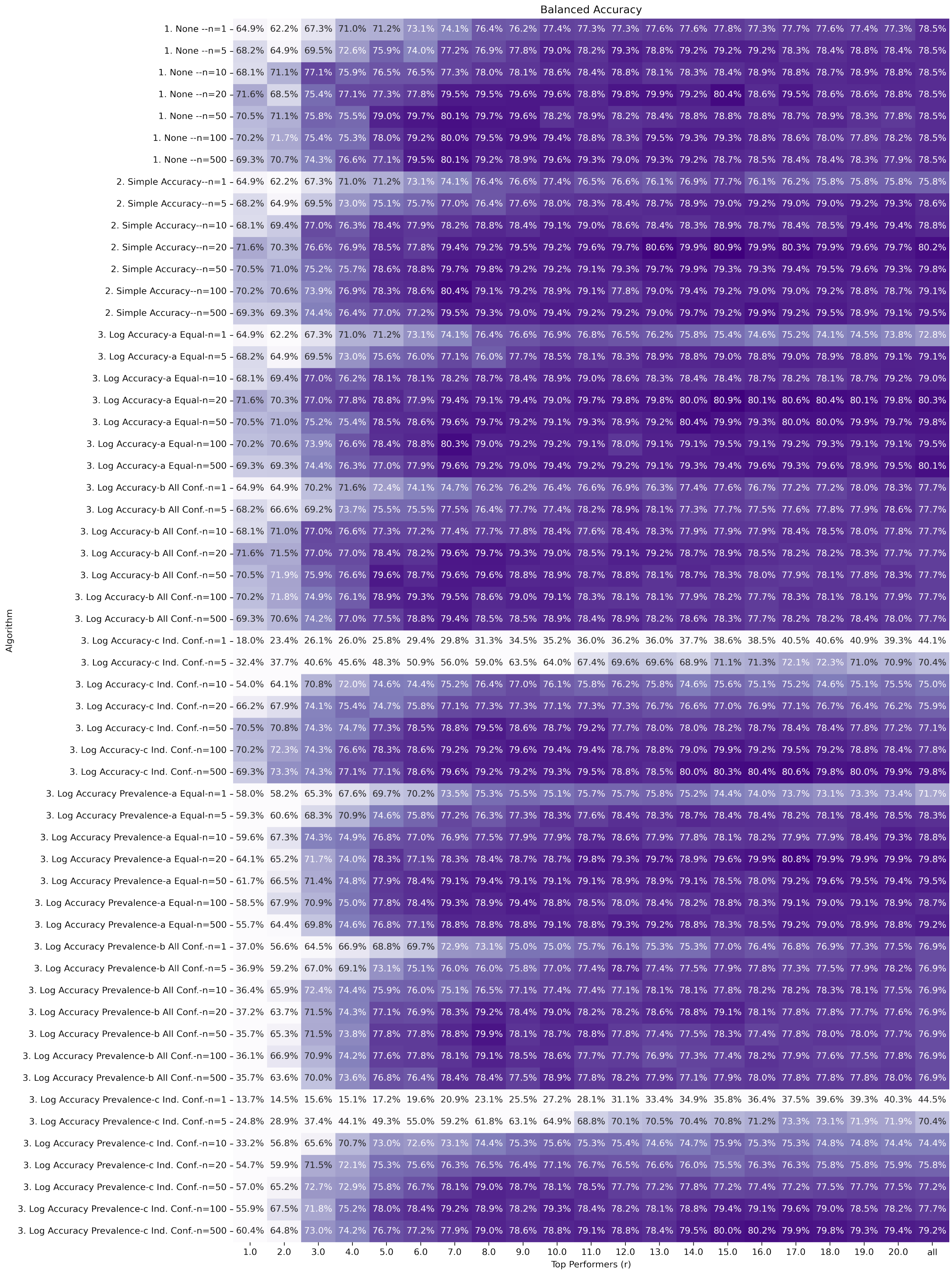

Figure 9

Estimating the balanced accuracy of different algorithms while varying the number of decisions (n) used to estimate individual parameters.

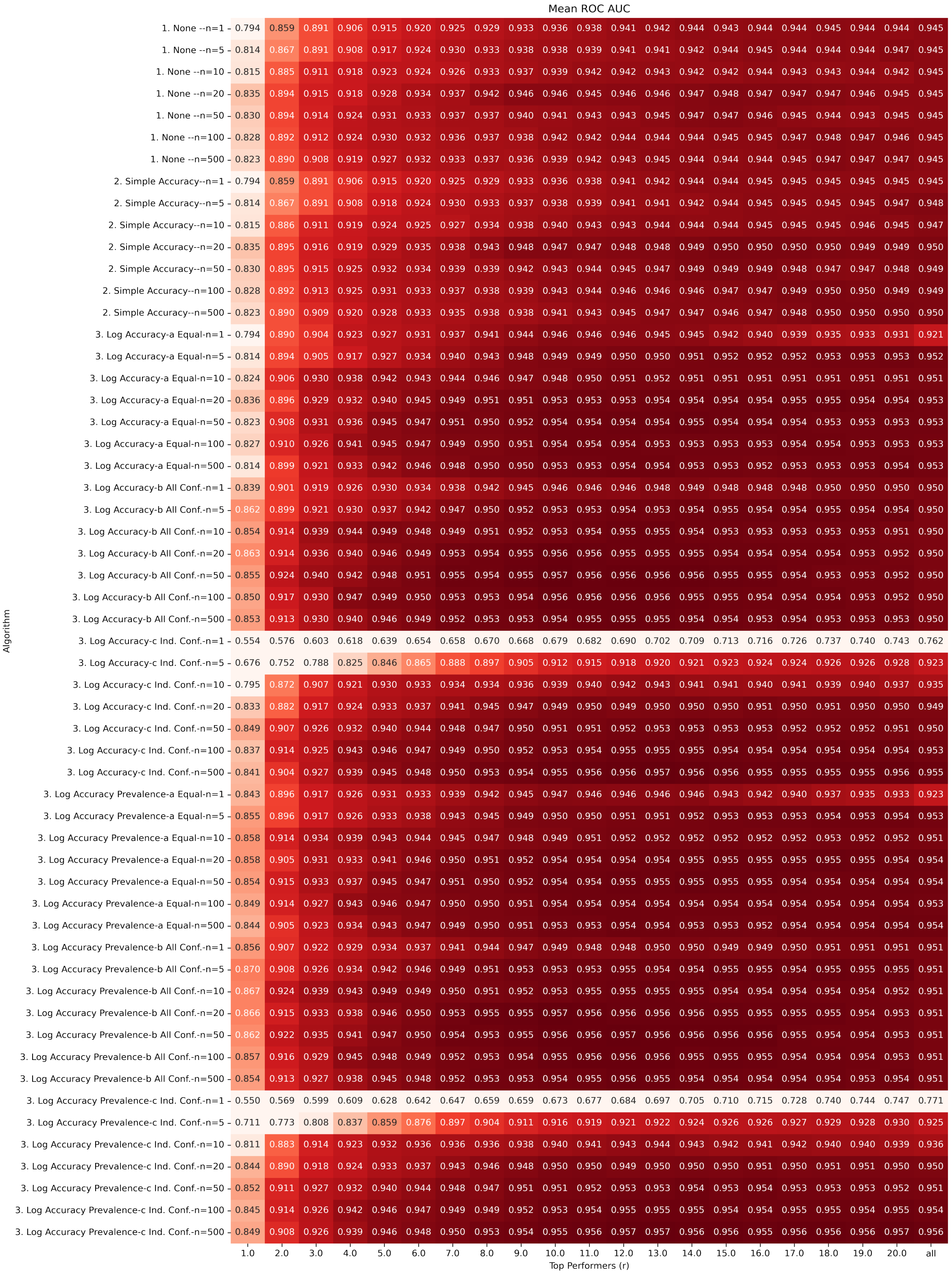

Figure 10

Estimating the mean ROC-AUC of different algorithms while varying the number of decisions (n) used to estimate individual parameters.

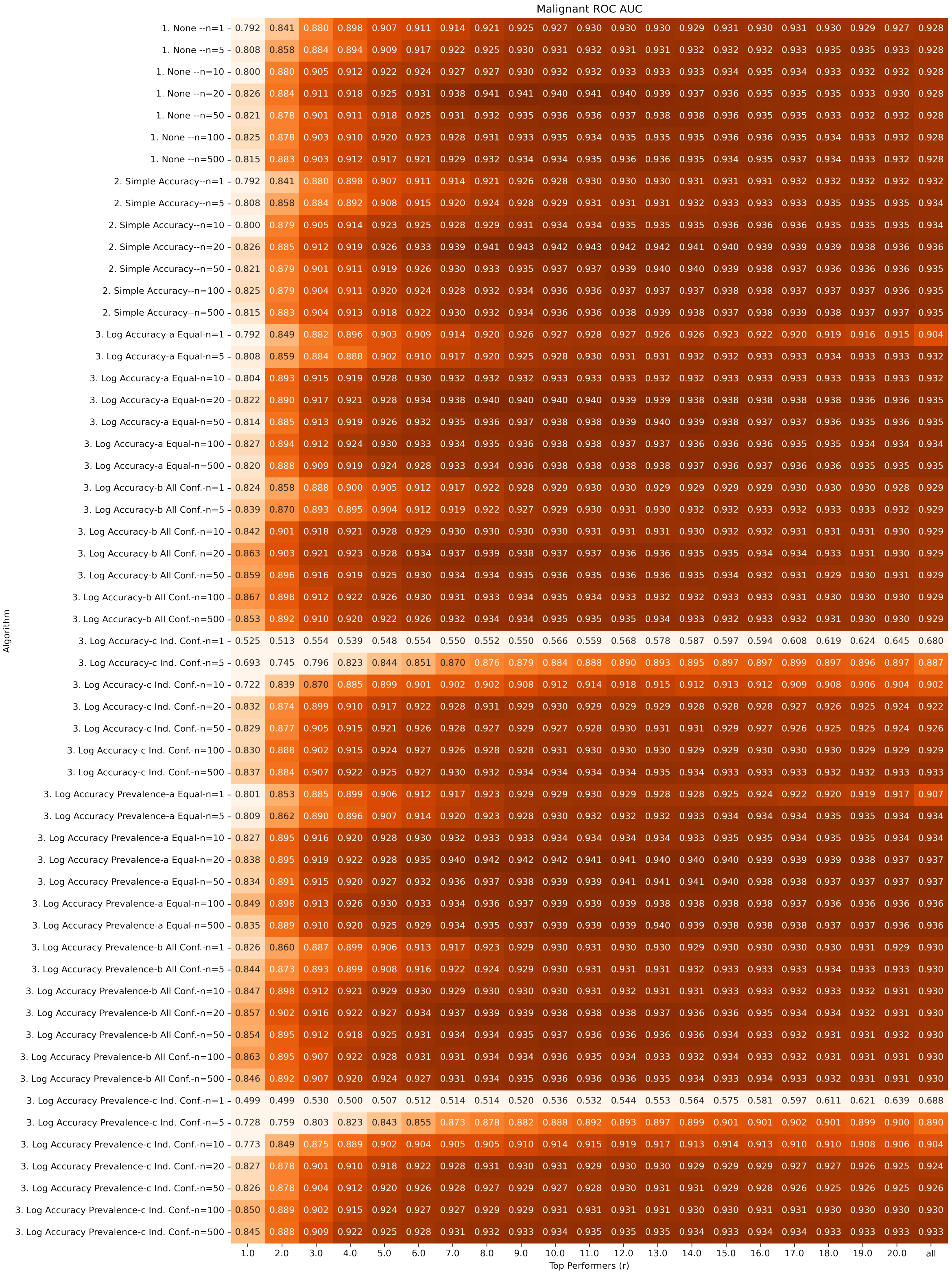

Figure 11

Estimating the malignant ROC-AUC of different algorithms while varying the number of decisions (n) used to estimate individual parameters.

Robustness of threshold

We vary the τ parameter that controls how to deal with very low or very high probabilities for the algorithms involving the log probability weighting. We observe that the performance of the algorithms do not depend on τ except for when it is very large. We test the values 0.01, 0.02, 0.05, 0.1 and 0.3 in the supplement. We present the results for $\tau = 0.02$ in the main paper.

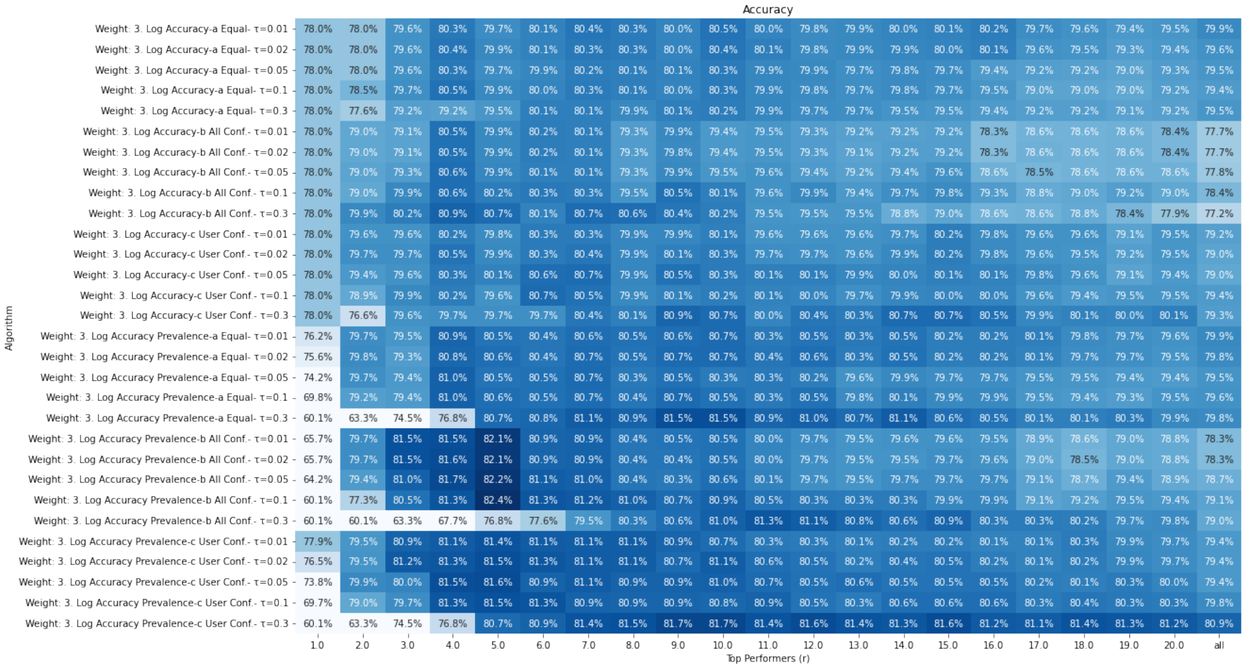

Figure 12

Accuracy - A robustness check of the threshold parameter in the accuracy based weighting algorithms that use the log weighting. The value of the threshold parameter (τ) is the last number in the left-hand side label.

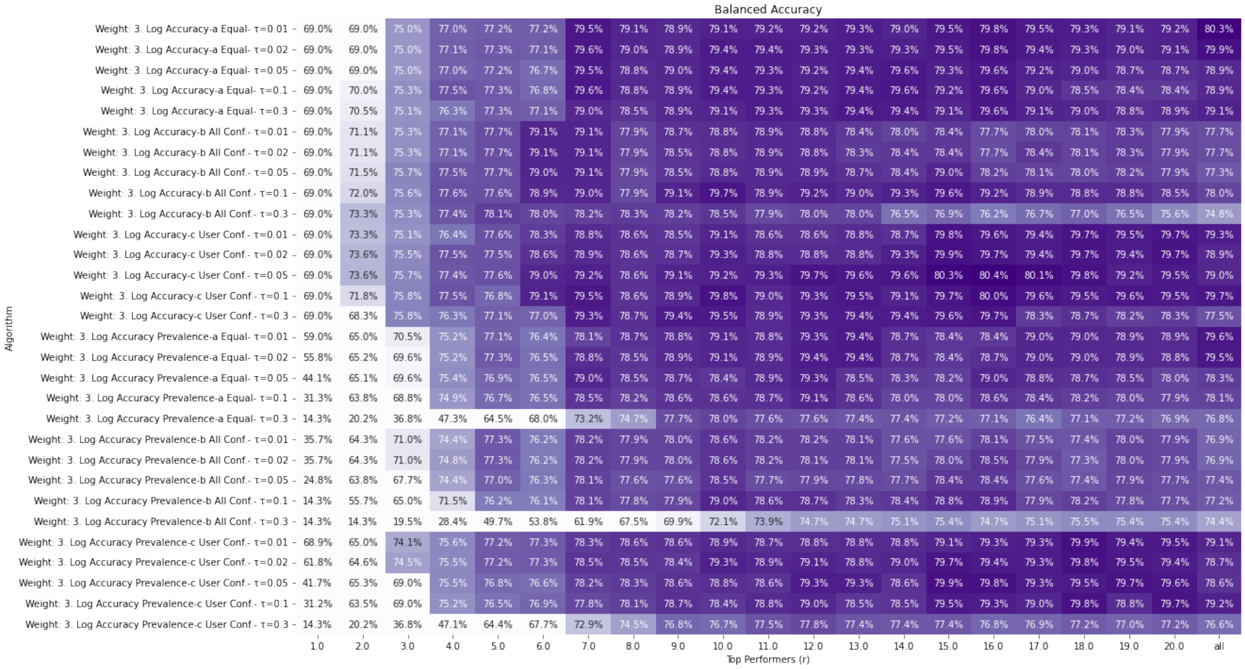

Figure 13

Balanced Accuracy - A robustness check of the threshold parameter (τ) in the accuracy based weighting algorithms that use the log weighting.

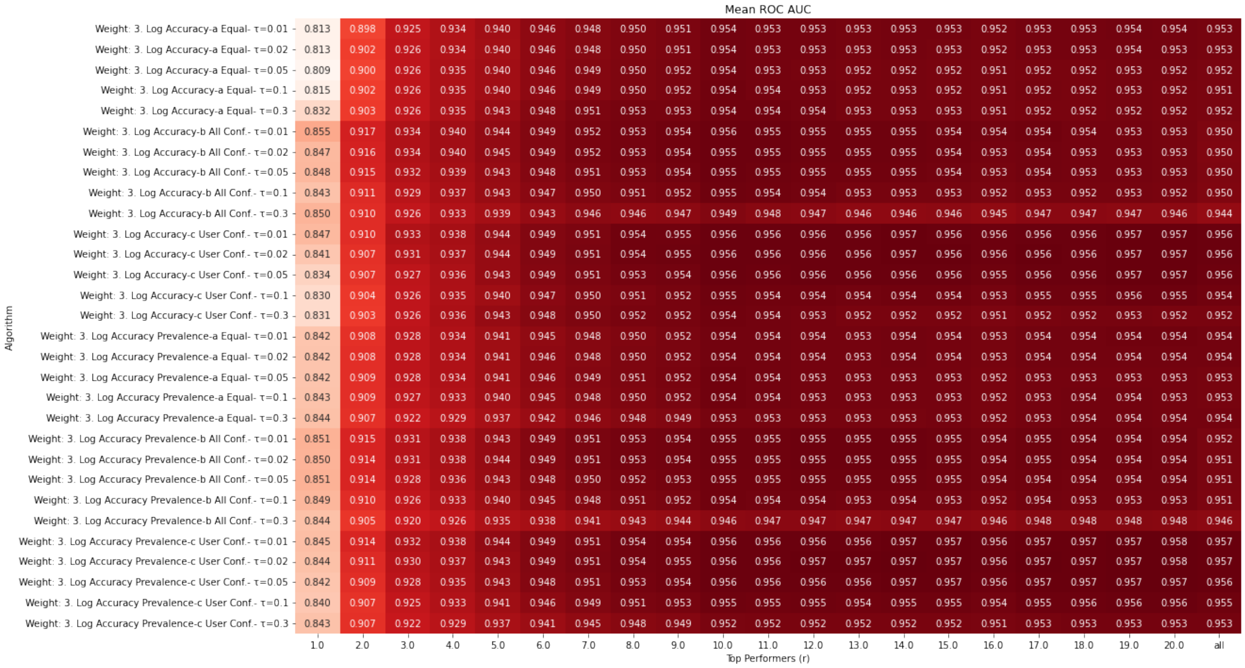

Figure 14

Mean ROC AUC - A robustness check of the threshold parameter (τ) in the accuracy based weighting algorithms that use the log weighting.

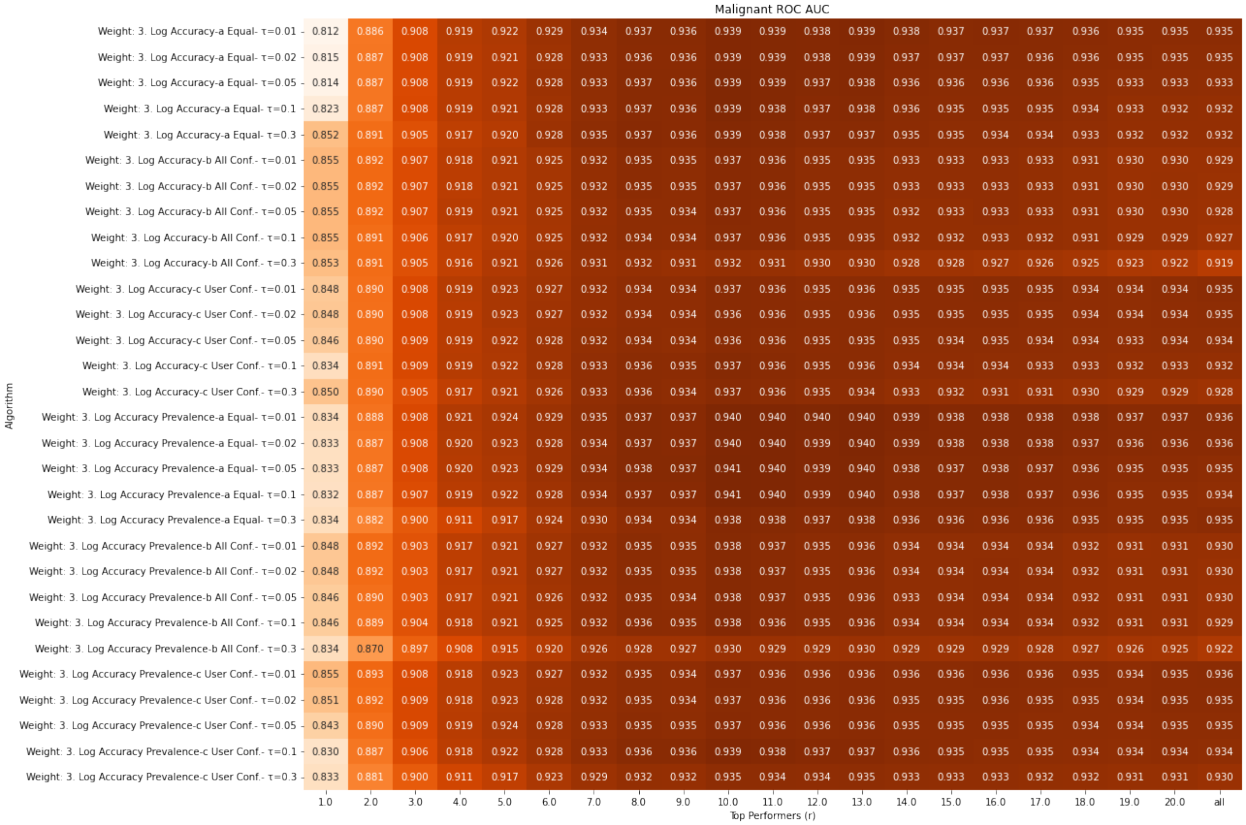

Figure 15

Malignant ROC AUC - A robustness check of the threshold parameter (τ) in the accuracy based weighting algorithms that use the log weighting.

Using individuals with a large number of responses

Demographics of High Response Individuals

Many algorithms that we tested involved estimating different parameters for each participant. We wanted to test if our results would change if we only used the individuals for whom we had better estimates of accuracy. Hence, we estimate these weights for the 51 individuals who made 500 decisions or more on the training data, which constitutes 72.98% of the train set. We conduct the switchboard analysis described in the main paper.

Since the demographics of the individuals who provided many responses might be different from the general population, we compiled the demographics of the individuals who provided more than 500 training responses in Tables 3, 4 and 5.

We present the results of our switchboard analysis in Figure 16

Gender	Number	Percentage
Male	25	55.56%
Female	18	40.00%
Other	2	4.44%

Table 3

Gender Distribution of the Individuals that provided more than 500 train responses

Occupation	Number	Percentage
No Medical Experience	2	4.35%
Pre-Med Student	4	8.70%
Medical Student	28	60.87%
Resident Or Fellow	2	4.35%
Attending Physician	2	4.35%
Registered Nurse	1	2.17%
Nurse Practitioner	1	2.17%
Other Medical Professional	5	10.87%

Table 4

Occupational Distribution of the Individuals that provided more than 500 train responses.

Experience	Number	Percentage
0 years	26	57.78%
<1 year	7	15.56%
1-3 years	6	13.33%
3-5 years	3	6.67%
5-10 years	1	2.22%
10+ years	2	4.44%

Table 5

Experience Distribution of the Individuals that provided more than 500 train responses.

Region	Number	Percentage
Americas	19	43.18%
Asia	18	40.91%
Europe	4	9.09%
Africa	3	6.82%

Table 6

Regional Distribution of Individuals that provided more than 500 train responses.

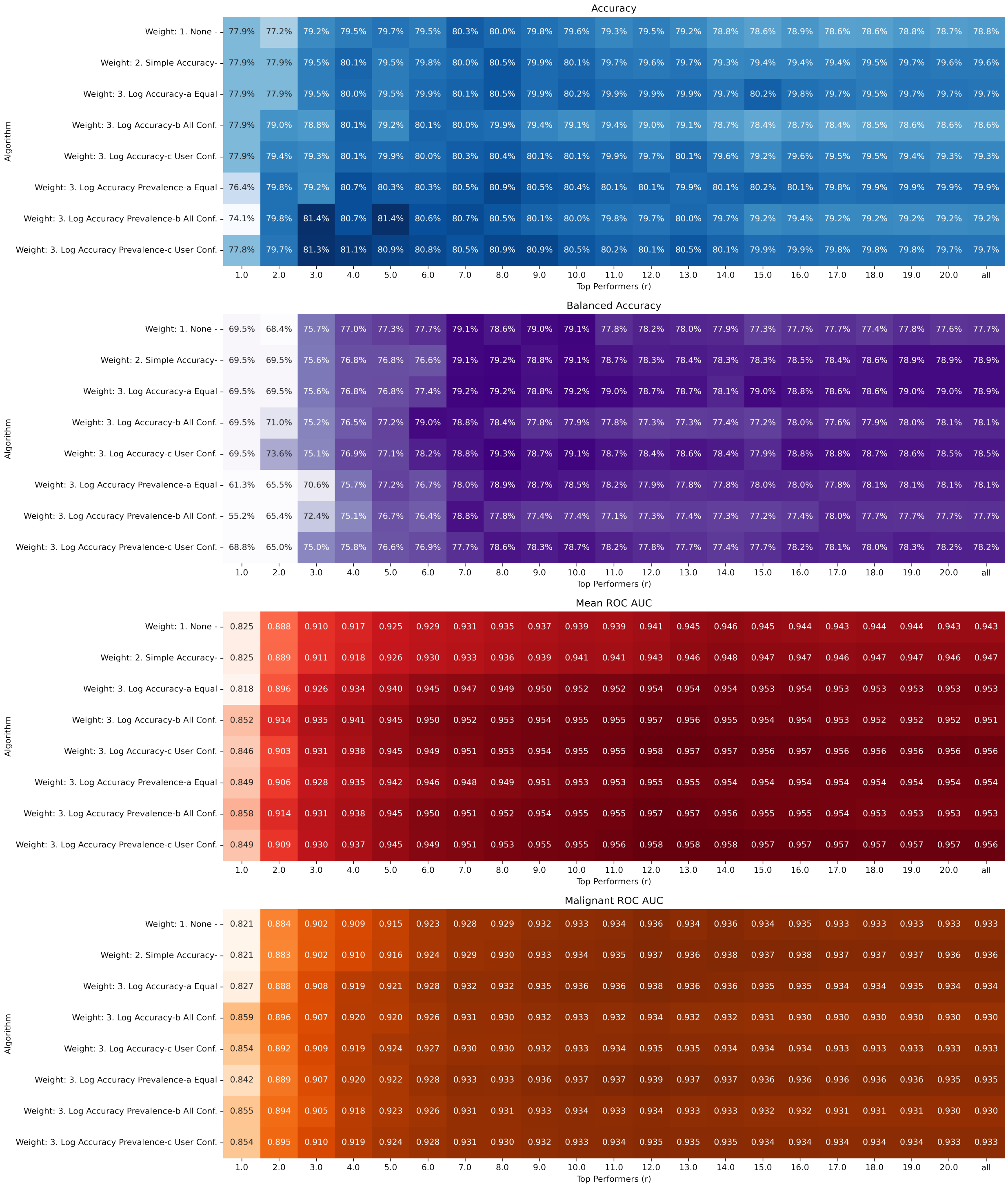

Figure 16

Switchboard analysis using the decisions made by users with more than 500 train images.
